# Discovery and preclinical evaluation of monoclonal antibodies and bispecific engagers targeting the NKG2A inhibitory receptor

**DOI:** 10.1126/sciadv.adu0690

**Published:** 2026-02-04

**Authors:** Seungmin Shin, Yae-Jin Kim, Bernard J. C. Macatangay, Joshua C. Cyktor, Margaret G. Hines, Ze-Yu Sun, Kong Chen, John W. Mellors, Dimiter S. Dimitrov, Wei Li, Du-San Baek

**Affiliations:** ^1^ Division of Infectious Diseases, Department of Medicine, University of Pittsburgh School of Medicine, Pittsburgh, PA, USA.; ^2^ GLPG US, 1401 Forbes Avenue, Pittsburgh, PA, USA.; ^3^ Division of Pulmonary, Allergy, and Critical Care Medicine, Department of Medicine, University of Pittsburgh, Pittsburgh, PA, USA.

## Abstract

NK and T cells are key effectors that eliminate cancer cells, but upregulation of the inhibitory receptor NKG2A on these cells attenuates antitumor immune responses. To counteract NKG2A inhibitory signaling, we identified two specific fully human monoclonal anti-NKG2A antibodies that block HLA-E ligand binding. These antibodies activated NK cells and enhanced antibody-dependent cellular cytotoxicity of tumor-targeting IgG1s both in vitro and in vivo. Bispecific engagers (BiNKs), generated by fusing NKG2A antibodies with tumor targeting binders, promoted immune synapse formation and directed cytotoxicity of NK and CD8^+^ T cells toward cancer cells. In a human PBMC-engrafted NSG mouse xenograft lung cancer model, an anti-HER2 × anti-NKG2A BiNK markedly inhibited tumor growth as a monotherapy or in combination with pertuzumab. Cell depletion studies revealed that the BiNK enhanced antitumor activity of both NK and T cells. NKG2A blockade with potent and specific, fully human antibodies and BiNKs show promise for further development as cancer immunotherapeutics.

## Teaser

NKG2A antibody blockade and bispecific NKG2A engagers enhance NK and T cell antitumor activity.

## INTRODUCTION

The clinical success of immune checkpoint inhibitors (ICIs) has ushered in a new era of cancer immunotherapy, expanding therapeutic options for patients across a variety of cancers. Monoclonal antibodies (mAbs) targeting programmed cell death protein–1 (PD-1) and programmed cell death ligand-1 (PD-L1) have provided unprecedented and durable clinical benefits in a subset of patients (*1*–*3*). Nevertheless, a substantial proportion of patients fail to respond to anti–PD-1/PD-L1 therapy, highlighting the need to develop alternative or complementary immunotherapeutic strategies (*4*–*7*).

One alternative strategy currently under investigation is the blockade of additional inhibitory pathways on T cells and natural killer (NK) cells that dampen immune responses. NKG2A, an inhibitory receptor of the NKG2 family, has emerged as a promising target to block as a complement to existing immunotherapies (*8*–*10*). NKG2A forms a heterodimer with CD94 and is predominantly expressed on NK cells, NK T (NKT) cells, and a subset of CD8^+^ T cells. The engagement of the CD94/NKG2A heterodimer with its ligand, the nonclassical major histocompatibility complex (MHC) class I molecule human leukocyte antigen (HLA)–E, triggers phosphorylation of the immunoreceptor tyrosine-based inhibitory motifs (ITIMs) in the cytosolic domain of NKG2A (*11*, *12*). This event recruits the Src homology 2 (SH2) domain-containing tyrosine phosphatases SHP-1 and SHP-2, which subsequently dephosphorylate and inactivate critical signaling molecules such as VAV1, ZAP70, and members of the Src family kinases involved in NK cell activation pathways mediated by activating receptors including NKG2C and D, thereby suppressing NK cell cytotoxic activity. The elevated expression of HLA-E on the surface of cancer cells or HIV-infected lymphocytes has been reported, whereas NK cells lacking NKG2A eliminate these target cells (*13*–*16*). Similarly, the inhibition of NKG2A-mediated signal transmission by an anti-NKG2A monoclonal antibody, monalizumab (humanized Z270 murine mAb), restores NK and T cell effector function in preclinical cancer models (*10*, *13*, *17*). Consequently, monalizumab is now being studied in multiple clinical trials (NCT04307329, NCT04590963, NCT05221840, and NCT05061550) to increase the proportion of patients responding to ICI. The combination of monalizumab with the anti–PD-L1 antibody, durvalumab, has led to encouraging responses in patients with unresectable Stage III non–small cell lung cancer (NSCLC) and is being evaluated in a phase 3 registrational trial (*18*).

In search of more potent and specific inhibitors of NKG2A, we report here the discovery of a fully human anti-NKG2A–specific mAb designated as 1B2. Through affinity maturation using yeast display technology, we obtained an affinity-enhanced clone (1B2-6) that exhibits markedly improved blockade of HLA-E binding to the CD94/NKG2A heterodimer. Both 1B2 and 1B2-6 partially activated primary NK cells from healthy donor peripheral blood mononuclear cell (PBMC) and promoted NK cell–mediated antibody-dependent cellular cytotoxicity (ADCC) of cancer cells in combination with immunoglobulin G1 (IgG1) antibodies targeting tumor antigens including epidermal growth factor receptor (EGFR), human epidermal growth factor receptor 2 (HER2), and carcinoembryonic antigen-related cell adhesion molecule 5 (CEACAM5). Building from these results, we constructed and evaluated first-in-class bispecific engagers targeting NKG2A (BiNK) and tumor-specific antigens, which elicit robust NK and CD8^+^ T cell–mediated cancer cell cytotoxicity both in vitro and in vivo. Mechanistic analyses revealed that formation of an immune synapse (IS) between NK and cancer cells, blockade of the NKG2A inhibitory pathway, and potential induction of NKG2A receptor internalization collectively contribute to BiNK-induced cancer cell killing.

## RESULTS

### The 1B2 clone differentiates between NKG2A and NKG2C and inhibits HLA-E interaction

**Fig. 1.**
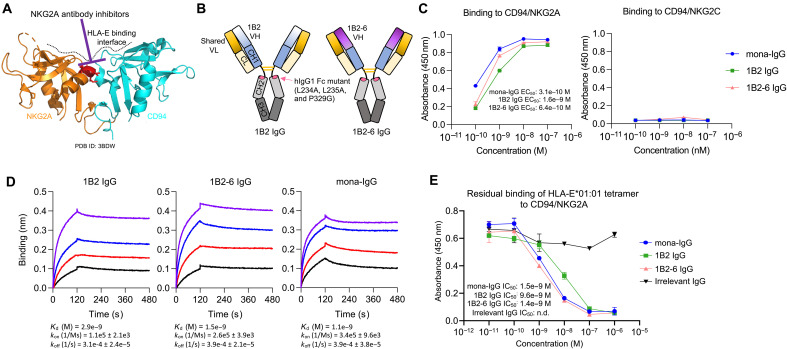
Discovery of NKG2A binding antibodies, 1B2 and 1B2-6. (**A**) Crystal structure of CD94/NKG2A (PDB ID: 3BDW). Hypothetical critical epitope for 1B2, 1B2-6, and mona-IgG is highlighted as red in both structures. (**B**) Schematic figure depicting components of 1B2 and affinity matured 1B2-6 human IgG1 having L234A, L235A, and P329G mutations to abolish binding ability to Fc gamma receptors. (**C**) Binding of 1B2, 1B2-6, and mona**-**IgG to CD94/NKG2A or CD94/NKG2C by ELISA. (**D**) Binding of 1B2, 1B2-6, and mona-IgG to CD94/NKG2A by biolayer interferometry (BLI). (**E**) Competitive ELISA result with sHLA-E tetramer demonstrating affinity matured 1B2-6 IgG has improved blocking capability compared to parent 1B2 IgG. ELISA results are representative of two replicates, and data are presented as means ± SD. EC_50_, median effective concentration; n.d., not determined.

NKG2A shares a high homology with the activating receptor NKG2C (amino acid homology >90%, fig. S1A). We adopted a competitive phage panning strategy to isolate NKG2A-specific binders from an in-house fully human Fab phage library using NKG2A as the panning bait and NKG2C as the counter-selection competitor. We produced recombinant NKG2A and NKG2C proteins fused with human IgG1 Fc. Because NKG2A forms a heterodimer with CD94 and HLA-E binds to the NKG2A/CD94 interface (Fig. 1A), we also produced the NKG2A/CD94 complex as the panning reagent (fig. S1B). A seven-residue linker (GSGGSGG) was used to fuse CD94 with NKG2A based on the crystal structure analysis of the CD94 and NKG2A heterodimer [Protein Data Bank (PDB) ID: 3BDW]. To assess the epitope exposure of these recombinant NKG2 proteins, we generated a mirroring clone of monalizumab, named mona-IgG, which bears L234A, L235A, and P329G (LALA-PG) mutations in the human IgG1 Fc region to abolish effector function instead of the IgG4 isotype in monalizumab, which is in clinical development. Only the CD94/NKG2A heterodimer complex bound to the mona-IgG, while NKG2A alone and NKG2C proteins showed no binding (fig. S1C), indicating that CD94 is an essential partner to generate the monalizumab binding epitope. Consequently, we used CD94/NKG2A heterodimer complex as the panning antigen to successfully identify two Fab clones, 1B2 and 2A8, that selectively bind to CD94/NKG2A but not NKG2C by enzyme-linked immunosorbent assay (ELISA) (fig. S1D).

We then investigated whether two Fabs could bind to natural CD94/NKG2A heterodimer on the membrane of primary human NK cells in PBMC from healthy donors. Flow cytometric analyses showed that the 1B2 Fab bound to primary human NK cells with a similar detection positivity (~20% after subtracting the secondary antibody signal) as mona-IgG (fig. S1E). Moreover, 1B2 binding is specific since it did not bind to NKG2A-negative B lymphocyte cell line (Farage) (fig. S2E). By contrast, we did not observe binding to CD94/NKG2A on human NK cells by a different Fab 2A8 (fig. S1E). We therefore excluded 2A8 from further investigation. We reformatted the 1B2 Fab into IgG with the LALA-PG mutation in Fc (named 1B2 IgG) (Fig. 1B) and assessed the capacity of 1B2 to block HLA-E ligand binding to its cognate receptors CD94/NKG2A and CD94/NKG2C. The competitive ELISA showed that 1B2 IgG inhibited the binding of soluble HLA-E tetramer (sHLA-E*01:01 with peptide sequence VMAPRTLVL) to CD94/NKG2A in a concentration-dependent manner (fig. S1F). 1B2 IgG is less potent than mona-IgG for blocking ligand, as reflected by its ~20-fold higher half-maximum inhibitory concentration (IC_50_). Furthermore, 1B2 IgG and mona-IgG did not decrease the binding of sHLA-E tetramer to CD94/NKG2C, indicating that these two IgGs bind to NKG2A-specific epitopes that differentiate between NKG2A and NKG2C (fig. S1F).

### Affinity maturation by yeast display yielded the more potent anti-NKG2A 1B2-6 clone

Although 1B2 IgG inhibited sHLA-E tetramer binding to NKG2A, the IC_50_ of 1B2 IgG (fig. S1F) indicated that higher affinity was needed for blocking of HLA-E binding comparable to mona-IgG. We designed and constructed a single chain Fab (scFab) yeast library for affinity maturation of 1B2 (fig. S2A) and separately optimized variable domain of the heavy chain (VH) and variable domain of the light chain (VL) sequences to obtain affinity-enhanced clones. To perform the affinity maturation of the VH domain, we replaced 1B2 VH with naïve VHs derived from healthy donor B cell repertoires, except for CDR-H3 and FR4, to avoid major epitope shifting. For VL, we shuffled the whole 1B2 VL to naïve VLs. We then monitored the expression and binding of scFabs displayed on the yeast surface and sorted out mixed yeast library cells that showed improved binding by flow cytometry (fig. S2B). One dominant clone, named 1B2-6, was identified from the library with VL shuffling. 1B2-6 exhibited enhanced binding affinity (~2-fold) to NKG2A/CD94 (Fig. 1, C and D, and fig. S2D) and improved the blocking of sHLA-E tetramer to NKG2A, with a similar IC_50_ as mona-IgG (Fig. 1E). We then investigated which residues in NKG2A are involved in selective binding of the three NKG2A antibodies (mona-IgG, 1B2, and 1B2-6 IgG) to NKG2A versus NKG2C. At the amino acid sequence level, three residues at positions 167, 168, and 170 differ among human NKG2 family proteins (A, B, C, E, and H) and in other species, except for chimpanzee (fig. S3, A and B). In addition, these three residues are located at the center position for the interaction with HLA-E based on the crystal structure of CD94/NKG2A and HLA-E complex (Fig. 1A), whereas other neighboring residues are almost identical between NKG2A and NKG2C. We hypothesized that one or two residues play critical roles as binding epitopes for NKG2A-specific antibodies. We then constructed three mutant proteins of NKG2A (S167A, I168S, and S170L) and tested their binding to the three antibodies. ELISA results revealed that the serine-170 (S170) of NKG2A is critical for binding all three NKG2A-specific antibodies (fig. S3C), although the substitution to the NKG2C counterpart did not lead to complete loss of binding, indicating some involvement of other residues.

### Activation of primary human NK cells by NKG2A blockade antibodies

**Fig. 2.**
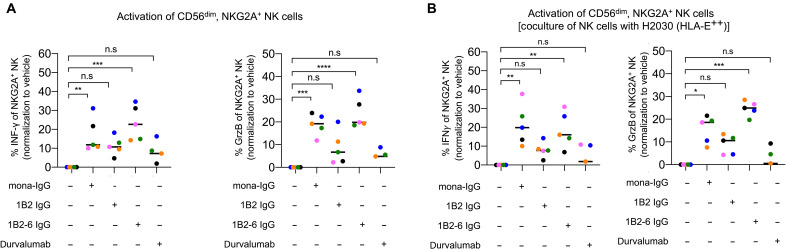
In vitro NK cell activation by anti-NKG2A antibodies. (**A** and **B**) Percentage of IFN-γ and GrzB-positive CD56dim/NKG2A^+^ NK cells to monitor NK cell activation in baseline NK cell alone (A) or cocultured with H2030 cancer cells (B) after treatment with NKG2A antibody and anti–PD-L1 antibody, durvalumab, for 24 hours. Each donor is represented by a single dot. The values were normalized to vehicle control. Significance was determined by one-way analysis of variance (ANOVA) with the Tukey’s post hoc test, *P < 0.05, **P < 0.01, and ***P < 0.001; n.s, not significant.

Resting NK cells are typically prearmed with basal activating programs such as constitutively transcribing and expressing cytotoxic effector genes and preforming cytolytic granules (*19*, *20*), allowing them to respond rapidly to infected or transformed cells. To avoid damage to normal “self” tissues, basal activation modules must be tightly constrained by continuous inhibitory signaling input by receptors such as NKG2A, through a process called NK cell education (*21*). We therefore sought to evaluate whether NKG2A blocking antibodies can activate NK cells. We performed experiments either at baseline or after incubation with HLA-E^+^ tumor cells. NK cell activation was monitored by interferon-γ (IFN-γ) and granzyme B (GrzB) secretion. IFN-γ is a master effector cytokine for NK function, and its induction requires engagement of a set of activating receptor signaling (*22*), while GrzB is an effector molecule for cytolysis. As shown in Fig. 2A and fig. S4 (A and B), treatment with anti-NKG2A antibodies activated CD56^dim^NKG2A^+^ primary NK cells, as manifested by increased expression of IFN-γ and GrzB. The 1B2-6- and mona-IgG exhibited similarly robust activation levels (>10% of cells). At the same time, 1B2 IgG only showed a tendency to activate without statistical significance, which is consistent with the lower HLA-E blocking capacity of 1B2. The anti–PD-L1 antibody, durvalumab, showed minimal activation. The activation of NK cells by these NKG2A blocking antibodies was recapitulated when cocultured with HLA-E–expressing NSCLC cell H2030 (Fig. 2B and fig. S4, C and D). However, coincubation with HLA-E^+^ tumor cells did not augment NK cell activation by NKG2A blocking antibody, indicating limited innate NK/tumor cell recognition.

The NKG2A/HLA-E signaling axis suppresses NK activation mainly through two distinct mechanisms: the SHP-1/2–dependent pathway and the Crk phosphorylation-dependent pathway. In the former, HLA-E binding leads to phosphorylation of NKG2A intracellular ITIM, followed by recruitment of SHP-1 and SHP-2 (*23*), which dephosphorylate Vav1 (*24*). pVav1 is a master intermediate for NK cell activation that mediates Ca^2+^ mobilization, microtubule-organizing center (MTOC) organization, and F-actin remodeling (*25*, *26*). To investigate the underlying mechanism, we examined intracellular signaling events following 1B2-6 treatment by Western blotting (WB). WB data showed variation across three donors. From the merged data, we did not observe a reduction in phospho–SHP-1 levels following NKG2A blockade (fig. S5), consistent with the findings of Gong *et al.* (*27*), showing that the NKG2A antibody Z199, recognizing the same epitope as monalizumab, did not decrease phospho–SHP-1 (pSHP-1) levels either at baseline or after cancer cell coincubation. However, we detected a trend toward reduced pCrk and increased pVav1 (fig. S5), consistent with NK cell activation. These results suggest that 1B2-6 may modulate NKG2A signaling through an SHP-1–independent c-Abl/pCrk axis (*26*). However, we cannot exclude that our experiments were not sensitive enough to capture potential changes in pSHP-1.

### NKG2A blocking antibodies promote ADCC of tumor antigen-specific IgG1 antibodies

**Fig. 3.**
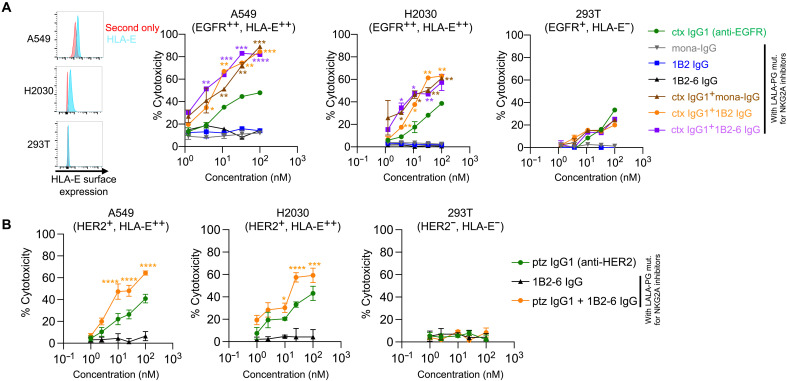
The enhanced ADCC effects of tumor-binding antibodies by combination with NKG2A antibodies in vitro. (**A**) Cell surface HLA-E expression level of A549, H2030, and 293T cells (left). ADCC activity of anti-EGFR hIgG1 cetuximab (ctx) with mona-IgG, 1B2 IgG, or 1B2-6 IgG in NSCLC A549, H2030 cells, and 293T cells (right graphs). (**B**) ADCC activity of anti-HER2 IgG1 pertuzumab with 1B2-6 IgG in NSCLC A549, H2030 cells, and 293T cells. (A and B) Lactate dehydrogenase (LDH) release assay results in presence of primary NK cells from healthy donor PBMCs. E:T ratio, 5:1. Significance was determined by unpaired two-tailed Student’s *t* test. *P < 0.05, **P < 0.01, ***P < 0.001, and ****P < 0.0001 versus ctx or pertuzumab (ptz) single treatment in vitro. (A) Cell surface HLA-E expression level of A549, H2030, and 293 T cells (left). ADCC activity of anti-EGFR hIgG1 cetuximab (ctx IgG1) with mona-IgG, 1B2 IgG, or 1B2-6 IgG in NSCLC A549, H2030 cells, and 293T cells (right graphs). (B) ADCC activity of anti-HER2 IgG1 pertuzumab (ptz IgG1) with 1B2-6 IgG in NSCLC A549, H2030 cells, and 293T cells. (A and B) LDH release assay results in presence of primary NK cells from healthy donor PBMCs. E:T ratio of 5:1. Significance was determined by unpaired two-tailed Student’s *t* test. *P < 0.05, **P < 0.01, ***P < 0.001, and ****P < 0.0001 versus ctx or ptz single treatment.

Tumor binding IgG1 antibodies recruit CD16A and promote formation of strongly activating ISs between NK cells and tumor cells expressing targeting antigens. The involvement of inhibitory receptor NKG2A in the IS may compromise the activating signaling. Hence, NKG2A blockade may synergize with antitumor IgG1 antibodies to facilitate Fc/CD16A clustering. Previous studies have demonstrated that monalizumab promoted ADCC of the anti-EGFR IgG1 antibody, cetuximab (*13*). Thus, we investigated whether 1B2 or 1B2-6 IgG could also enhance ADCC by three IgG1 antibodies (EGFR targeting cetuximab, anti-HER2 pertuzumab, and an anti-CEACAM5 antibody, 1G9 IgG1) (*28*). Before conducting cytotoxicity assays, we confirmed that IgG1 antibodies to cancer cell targets with wild-type Fc regions bound robustly to FcγRs, whereas mona-IgG, 1B2, and 1B2-6 IgG with LALA-PG mutations had significantly reduced binding to FcγRs (fig. S6A). We observed that combination treatment of anti-NKG2A antibodies with cetuximab significantly increased ADCC activity against EGFR^+^/HLA-E^+^ NSCLC A549 and H2030 cells compared to cetuximab alone (Fig. 3A). This enhancement of ADCC activity by the anti-NKG2A antibodies appeared dependent on HLA-E expression since no enhancing effect was observed when targeting EGFR-positive but HLA-E–negative 293T cells (Fig. 3A). We validated this ADCC enhancing activity of anti-NKG2A antibody by testing another IgG1 antibody pertuzumab targeting HER2 on the same A549 and H2030 cells (Fig. 3B). In addition, the cotreatment of 1B2 or 1B2-6 IgG enhanced the ADCC elicited by the anti-CEACAM5 1G9 IgG1 against the CEACAM5^+^/HLA-E^+^ neuroendocrine prostate cancer cell line NCI-H660 compared to 1G9 monotherapy at high antibody concentrations (fig. S6B). By contrast, anti-NKG2A antibodies failed to potentiate the ADCC activity of 1G9 against CEACAM5-positive prostate cancer Du145-CEACAM5 cells, which exhibits lower levels of HLA-E expression compared to NCI-H660 (fig. S6B). Intriguingly, while 1B2 IgG exhibited lower potency in inhibiting HLA-E binding to NKG2A/CD94 than 1B2-6 IgG, it demonstrated a similar enhancing effect on ADCC as mona-IgG and 1B2-6-IgG. Although the three anti-NKG2A antibodies increased GrzB and IFN-γ production in NK cells (Fig. 2, A and B), they failed to mobilize NK cell–mediated cytotoxicity against cancer cells in the absence of IgG1 directed against tumor antigen (Fig. 3, A and B). Unlike cytokine induction, cytolytic activity requires a different set of events involving formation of IS by receptor/ligand engagement, activating receptor immunoreceptor tyrosine-based activation motif–dependent phosphorylation leading to pVav1 amplification and F-actin and microtubule remodeling to polarize and exocytose cytolytic granules (*29*), which are not induced by NKG2A blockade alone. Without formation of effective cytolytic IS, even coculturing with HLA-E–positive tumor cells, NKG2A blockade simply removes inhibition but cannot induce cytolysis. Together, these results indicate that the fully human NKG2A-specific antibodies, 1B2 and 1B2-6 IgG, can augment the ADCC activity of antibodies to tumor-associated antigens. Our results also showed that NKG2A blockade alone can induce NK cytokine expression but that is insufficient to promote NK-mediated tumor cell lysis.

### Bispecific engagers targeting NKG2A (BiNK) induce NK cell–mediated tumor cell killing through simultaneously targeting of NKG2A and tumor antigens

**Fig. 4.**
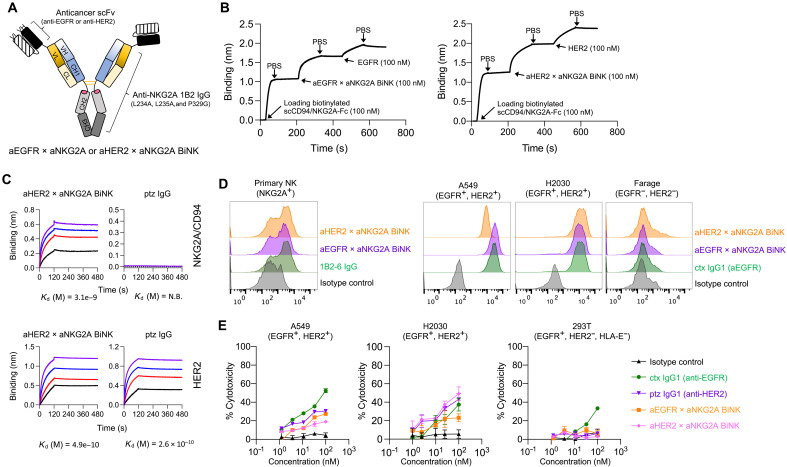
The target binding and cytotoxicity of BiNKs. (**A**) Schematic figure depicting the constructure of aEGFR × aNKG2A BiNK and aHER2 × NKG2A BiNK. (**B**) Graph showing the simultaneous binding of aEGFR × a NKG2A BiNK to EGFR and CD94/NKG2A-Fc (left) and aHER2 × NKG2A BiNK to HER2 and CD94/NKG2A-Fc (right). (**C**) Binding of aHER2 × aNKG2A BiNK and pertuzumab (ptz IgG1) to CD94/NKG2A or HER2 by BLI. N.B., no binding. (**D**) Cell surface binding of antibodies (100 nM) on the primary NK cells, A549, H2030, and Farage cells. (**E**) Cell killing activity of antibodies against A549, H2030, and 293T cells in the presence of NK cells (E:T ratio of 5:1) for 4 hours. Results are shown as the means ± SD for representative data from three independent experiments.

To induce NK cell cytotoxicity independent of ADCC, we hypothesized that bispecific NK cell engagers, simultaneously binding NKG2A on NK cells and surface antigens on cancer cells, could promote the formation of an IS by bringing these cells into proximity. These bispecific NKG2A-targeting engagers (BiNKs) represent a distinct approach from the current state-of-the-art NK cell engagers based on activating receptors such as CD16A and NKp46 (*30*). We engineered these BiNK molecules by fusing the single-chain variable fragment (scFv) derived from tumor-targeting antibodies (anti-EGFR or anti-HER2) to the N terminus of the light chain of anti-NKG2A antibody 1B2 IgG with LALA-PG mutations using a flexible GSGGGG polypeptide linker as a spacer (Fig. 4A). We produced BiNKs in expi293, and the BiNK protein displayed the expected size without oligomer formation in SDS–polyacrylamide gel electrophoresis (SDS-PAGE) and size exclusion chromatography (SEC) analyses (fig. S7, A and B). The biolayer interferometry (BLI) study showed that the anti (a)EGFR × anti (a)NKG2A BiNK simultaneously binds to the individual CD94/NKG2A-Fc and EGFR proteins (Fig. 4B). Similarly, aHER2 × aNKG2A BiNK simultaneously binds to the individual CD94/NKG2A-Fc and HER2 proteins. The binding dissociation constants (*K*_d_) of aHER2 × aNKG2A BiNK for binding to HER2 and NKG2A are similar as those of individual antibodies (Fig. 4C), indicating that the BINK bispecific molecular configuration does not negatively impact binding. We also confirmed the cell surface binding of BiNKs to NKG2A^+^ primary NK cells and EGFR^+^/HER2^+^ A549 and H2030 cells, whereas no binding was observed to the NKG2A^−^/EGFR^−^/HER2^−^ Farage cell line (Fig. 4D). Both aHER2 × aNKG2A and aEGFR × aNKG2A BiNKs were capable of inducing robust cytotoxicity mediated by NKG2A^+^ primary NK cells against the EGFR^+^/HER2^+^ tumor cell lines A549 and H2030 (Fig. 4E). The BiNK-elicited NK cell killing was specific and dependent on the expression of the targeted tumor antigens, as the aHER2 × aNKG2A BiNK failed to kill the EGFR^+^/HER2^−^ 293 T cells (Fig. 4E). Unlike what we observed in A549 cells, in which the tumor-binding IgG1 pertuzumab induced higher ADCC than the corresponding BiNK, we found similar efficacy for pertuzumab and BiNK in H2030 cells. Thus, it appears that BiNK activity may depend on the target tumor cells. Another confounding factor is the relative abundance of effector NK cell subsets in our assay. As shown in fig. S7C, the proportion of NKG2A^+^ NK cells among total CD56^+^ NK cells from peripheral blood is ~25%, whereas CD56^dim^ CD16A^+^ cytolytic NK cells comprise ~72% of total CD56^+^ NK cells. These proportions of NK cell subsets are consistent with what others have reported (*31*–*37*). Thus, under our assay conditions (total NK:tumor cell = 5:1), the effective effector-to-target (E:T) ratio for BiNKs is only ~1.3:1 compared with ~3.6:1 for IgG1 antibodies, which likely contributes to the lower overall cytolytic readout.

### Mechanistic studies of BiNK-mediated NK cell toxicity against target antigen-expressing tumor cells

**Fig. 5.**
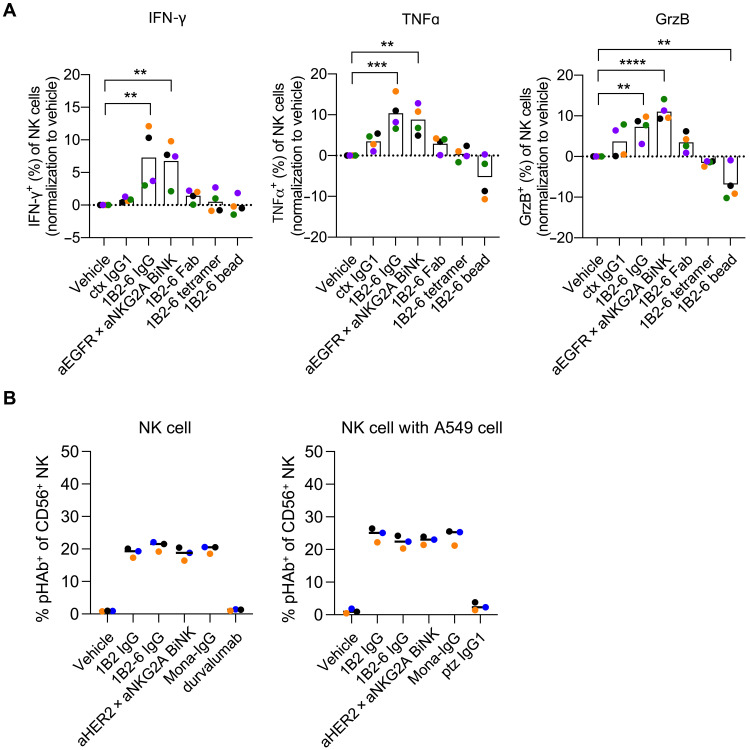
The NK cell activation through bivalent NKG2A binding and NKG2A down-regulation. (**A**) The IFN-⍺^+^, TNFα^+^, and GrzB^+^ population (%) of IL-2–activated NK cells after antibodies treatment with different NKG2A binding valency (monovalent, bivalent, tetravalent, or multivalent) in the coculture system with A549 cells, E:T ratio of 5:1, for 24 hours. (**B**) Internalization of NKG2A antibodies in NK cells, assessed by labeling the antibody with a pH-sensitive dye to monitor endocytosis. Durvalumab and pertuzumab (ptz IgG1) were included as controls. (A and B) Each symbol represents the value obtained from individual healthy donors. Significance was determined by one-way ANOVA with the Tukey’s post hoc test, *P < 0.05, **P < 0.01, ***P < 0.001, and ****P < 0.0001.

Next, we sought to explore the potential molecular mechanisms underlying the cytolytic activity of BiNKs. The aEGFR × aNKG2A BiNK failed to kill the EGFR^+^/HLA-E^−^ 293T cells but can kill the EGFR^+^/HLA-E^+^ A549 cells (Fig. 4E), suggesting that blocking NKG2A/HLA-E inhibitory signaling might contribute to BiNK activity. Our WB analysis showed that aEGFR × aNKG2A BiNK treatment slightly increased pVav1 (fig. S5), which is an important activating intermediate that helps to form the cytolytic IS (*25*), consistent with the BiNK-mediated induction of target cell lysis. Since activating signals are needed for pVav1 mobilization (*38*), the WB data imply that BiNKs can recruit activating receptors. We also observed that BiNK stimulated NK cells to secrete effector cytokines [IFN-γ and tumor necrosis factor (TNF)] and GrzB after coculturing with A549 tumor cells (Fig. 5A and fig. S8A). Cytokine release relies on the target cell recognition and activating receptor engagements (*22*).

Ligand-induced receptor oligomerization and subsequent activation depend on binding valency (*39*). We explored the impact of NKG2A binding valency on NK cell activation. We made and evaluated monovalent (1B2-6 Fab), bivalent 1B2-6 IgG, bivalent BiNK, tetravalent (1B2-6 SA mix), and multivalent (1B2-6 SA bead mix). We treated NK cells/A549 coculture with those formats and monitored the level of activating mediators. The results showed that the bivalent BiNK and 1B2-6 IgG efficiently activated NK cells secretion of IFNγ, TNF and GrzB, whereas the monovalent 1B2-6 Fab was insufficient to do that (Fig. 5A and fig. S8A). The tetravalent and multivalent 1B2-6 constructs failed to activate NK cells. The multivalent 1B2-6 even inhibited NK cell secretion of TNF and GrzB (Fig. 5A), consistent with the WB data showing increased pCrk and reduced pVav-1 for multivalent 1B2-6 (fig. S5), likely due to 1B2-6–coated beads serving as surrogate HLA-E^+^ cells, thereby activating the inhibitory NKG2A/CD94 pathway.

**Fig. 6.**
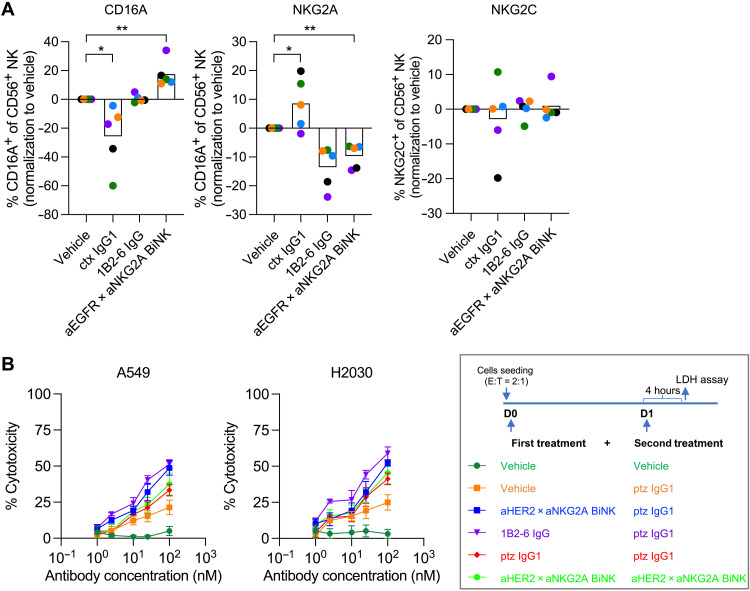
NK cell receptor modulation and functional activity induced by BiNK antibodies. (**A**) CD16^+^, NKG2A^+^, or NKG2C^+^ population (%) of CD56^+^ NK cells after antibodies (100 nM) and IL-2 treatment (50 IU/ml) in the coculture system with A549 cells, E:T ratio of 5:1, for 24 hours. (**B**) Left: Graphs showing the cell killing activity of aHER2 × aNKG2A BiNK cotreatment with anti-HER2 IgG1 pertuzumab (ptz IgG1) in A549 and H2030 cells in the presence of primary NK cells. E:T ratio of 2:1. Right: Schematic figure of antibodies treatment schedule. (A) Each symbol represents the value obtained from individual healthy donors. Significance was determined by one-way ANOVA with the Tukey’s post hoc test, *P < 0.05 and **P < 0.01.

The above results suggest that multivalent engagement of NKG2A by antibodies may paradoxically trigger, rather than block, inhibitory signaling. This raises the concern that BiNK, upon clustering through tumor-associated antigens, could potentially induce NKG2A-mediated inhibitory signaling. Although BiNK activated NK cells, BiNK treatment seemed increase pSHP-1 levels in our WB analysis (fig. S5), implying that BiNK-induced NK/tumor IS formation may promote NKG2A clustering and downstream pSHP-1 signaling. Nevertheless, BiNK must concurrently recruit activating receptor signaling within the synapse that drive NK cell activation that overrides the NKG2A inhibitory signal as manifested by increased pVav-1 levels and NK activation and target cell killing. To test this further, we phenotyped NK cells after coculture with EGFR^+^/HLA-E^+^ A549 tumor cells treated with either aEGFR × aNKG2A BiNK or 1B2-6 IgG. While neither BiNK nor 1B2-6 IgG affected the levels of NKG2C, BiNK significantly up-regulated the expression of CD16A, whereas 1B2-6 IgG had no effect (Fig. 6A and fig. S9A). We suspect that this CD16A upregulation may involve inhibition of a disintegrin and metalloproteinase 17 (ADAM17)-mediated CD16A shedding (*40*). In line with possible BiNK-mediated CD16A up-regulation, we found that aHER2 × aNKG2A BiNK promote ADCC activity of the pertuzumab IgG1 antibody against HER2^+^ A549 cells in a sequential combination treatment cytotoxicity assay, in which the cell cocultures were pretreated with BiNKs for 24 hours to allow for CD16A up-regulation, followed by treatment with tumor targeting IgG1 (Fig. 6B). We validated this observation on an additional cell line (H2030) (Fig. 6B). We confirmed that recombinant interleukin-2 (rIL-2) was not necessary for ADCC (fig. S9B). Although BiNK, but not 1B2-6, increased CD16A levels, BiNK did not promote more ADCC of pertuzumab IgG1 than 1B2-6, likely due to the negative impact of clustered BiNK-induced inhibitory signaling.

Antibody-driven target internalization and degradation have been extensively documented for cell surface receptors, such as HER2 (*41*, *42*). Antibody-induced receptor crosslinking and lattice formation promote endocytosis through either clathrin-dependent or caveolae-mediated pathways (*43*). The PD-1 antibody pembrolizumab has been reported to reduce cell surface levels of PD-1, serving as an additional mechanism to restore T cell functionality beyond blocking the PD-1/PD-L1 inhibitory axis (*44*). To determine whether our antibodies induce NKG2A internalization, we used a surrogate internalization assay to measure antibody uptake after binding. Similar approaches have been used to assess antibody-mediated CD73 internalization (*45*). This method is suitable for the antibodies studied here, as they contain the AAG mutation, which abolishes FcγR binding and therefore excludes Fc-mediated internalization. We conjugated the antibodies with a pH-sensitive fluorescent dye (pHAb) and monitored intracellular fluorescence in NK cells (*45*). Durvalumab and pertuzumab were included as negative controls. We incubated pHAb-conjugated antibodies with NK cells, either alone or in the presence of tumor cells A549. We observed robust fluorescence for all NKG2A antibodies (Fig. 5B and fig. S8B), indicating that upon binding to NKG2A, antibodies were internalized and trafficked into acidic intracellular compartments, such as endosomes and lysosomes. BiNK showed similar internalization extent as 1B2-6 IgG and 1B2-IgG and mona-IgG. The antibody-induced receptor internalization may represent an additional mechanism contributing to BiNK-mediated NK cell activation by reducing surface NKG2A expression. Collectively, these results indicate that facilitating formation of cytolytic IS between NK and tumor cells by recruiting activating receptors, partial blockade of the NKG2A inhibitory pathway, and induction of receptor internalization may contribute to the mechanisms underlying BiNK-induced cytolysis of cancer cells.

### NKG2A blockade antibodies and BiNK exhibited potent in vivo efficacy

**Fig. 7.**
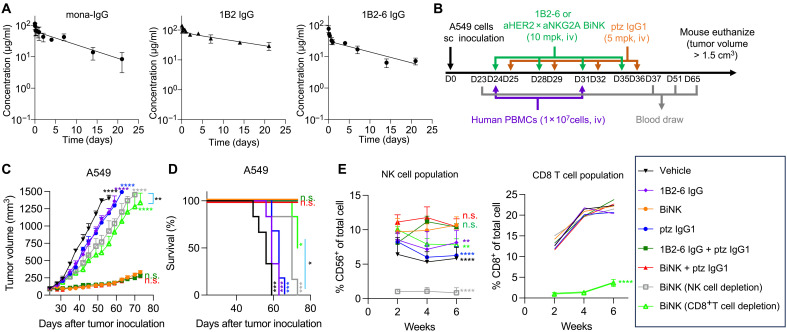
In vivo properties of NKG2A antibody inhibitors. (**A**) Serum concentration of mona-IgG, 1B2 IgG, and 1B2-6 IgG postantibodies injection (10 mg/kg) in female BALB/c mice. (**B**) Schematic figure of in vivo efficacy of anti-NKG2A antibody or aHER2 × aNKG2A BiNK or combination with anti-HER2 IgG1 pertuzumab (ptz IgG1) in A549 tumor xenograft NSG mice. (**C** and **D**) Tumor growth curve (C) and Kaplan-Meier survival curve (D) in repones to intravenous injection of the indicated antibodies. (**E**) Percentages of human NK cells (left) or CD8^+^ T cells (right) in mouse peripheral blood at the indicated time points. Blood was drawn at 2, 4, and 6 weeks for most groups except for vehicle at 5 weeks, 1B2-6 at 5.5 weeks, and pertuzumab (ptz) at 5.5 weeks. In [(C) and (E)], error bars represent the means ± SD (*n* = 6 per group). Significance of tumor growth inhibition and population of human NK or CD8^+^ T cell was determined by one-way ANOVA with the Tukey’s post hoc test, ****P < 0.0001 versus aHER2 × aNKG2A BiNK treatment group. Survival analysis was determined by a log-rank test, **P < 0.01 and ***P < 0.001 versus aHER2 × aNKG2A BiNK treatment group. Animals were euthanized when tumors reached ∼1.5 cm^3^ in size.

We evaluated the anti-tumor activity of the NKG2A antibody and BiNKs in a human PBMC-engrafted NOD-scid gamma (NSG) mouse xenograft model of lung cancer. We first examined the pharmacokinetic (PK) profile of anti-NKG2A antibodies by monitoring the serum concentration of mona-IgG, 1B2 IgG, and 1B2-6 IgG in BABL/c mice after intravenous injection (Fig. 7A). The terminal serum half-life (*T*_1/2_β) of mona-IgG, 1B2 IgG and 1B2-6 IgG were 7.0, 13.9, and 8.7 days, respectively. Both 1B2 IgG and 1B2-6 IgG exhibited typical biphasic clearance profiles and comparable serum half-lives to mona-IgG. Subsequently, we evaluated in vivo efficacy of anti-NKG2A antibodies, aHER2 × aNKG2A BiNK alone, and combination with the anti-HER2 IgG1 pertuzumab in an A549 lung tumor xenograft model (Fig. 7B). Previous studies have demonstrated that anti-NKG2A monoclonal antibodies promote antitumor immunity by enhancing both NK and CD8^+^ T cell responses (*13*). To dissect the contribution of each effector cell type, we included additional experimental groups in which human PBMCs were selectively depleted of NK cells or CD8^+^ T cells within the BiNK treatment cohorts. The results showed that aHER2 × aNKG2A BiNK alone, aHER2 × aNKG2A BiNK + pertuzumab, and 1B2-6+ pertuzumab combination groups effectively inhibited tumor growth, whereas pertuzumab or 1B2-6 alone showed minimal efficacy (Fig. 7C). In addition, BiNK alone or combination treatment significantly extended mouse survival (Fig. 7D). We found that the antitumor activity of aHER2 × aNKG2A BiNK alone depends on both NK and CD8^+^ T cells, as depletion of either cell population significantly compromised BiNK-mediated tumor inhibition (Fig. 7C). To support this result, we performed in vitro cytotoxicity assay using purified CD8^+^ T cells as effector cells, confirming that BiNK was able to directly activate CD8^+^ T cells to kill A549 tumor cells (fig. S10A).

Consistent with previous studies showing that NK cells have relatively short life span (several weeks) in the PBMCs humanized model due to lack of human cytokine (IL-15) support (*46*, *47*), our results showed NK cell number decreased with time for the inactive treatment groups (IgG 1B2-6 and pertuzumab alone groups) (Fig. 7E and fig. S10B). However, NK cell numbers were better maintained in the effective treatment groups (1B2-6 + pertuzumab, BiNK, and BiNK + pertuzumab), which may relate to the NK cell activation and persistence supported by the secreted cytokine milieu. By contrast, CD8^+^ T cell was expanded in all groups except the depletion group (Fig. 7E and fig. S10B), consistent with known xenoreactivity of human T cells recognizing murine MHC antigens (*48*); however, we did not observe any clinical signs of xenogeneic graft-versus-host disease, such as weight loss or organ dysfunction, during the study (fig. S10C). The T cell expansion reflects T cell activation in vivo, likely due to both tumor-specific and xenoreactive T cell responses. We concluded that the dual engagement of NKG2A^+^ NK and CD8^+^ T cells by the aHER2 × aNKG2A BiNK likely contributes to the in vivo antitumor efficacy observed.

## DISCUSSION

Immune checkpoint receptors have emerged as key targets for amplifying both cytotoxic NK cell and CD8^+^ T cell responses that mediate tumor regression and prolong survival. Following the remarkable clinical success of PD-1/PD-L1 blockades, NKG2A blockade also has been proposed as a next generation cancer immunotherapeutic. The elevated expression level of CD94/NKG2A, frequently observed in tumor-infiltrating lymphocytes, correlates with reduced survival in patients with cancer (*13*, *49*). The interaction between CD94/NKG2A heterodimer and its ligand HLA-E inhibits NK cell and T cell activation. Likewise, the aberrant expression of HLA-E on tumor cells has been implicated in cancer vaccine resistance, wherease the disruption of NKG2A-mediated inhibitory signaling by NKG2A antibodies potentiates vaccine-induced CD8^+^ T cell anticancer immune responses (*50*).

Monalizumab, the most extensively studied anti-NKG2A antibody has demonstrated anticancer activity in clinical trials and has advanced to later stages trials in combination with durvalamab. As a humanized antibody containing murine complementarity-determining regions (CDRs), it is susceptible to neutralization and accelerated clearance by the generation of antidrug antibodies targeting the murine sequences. In a phase 2 clinical study of patients with recurrent/metastatic squamous cell carcinoma of the head and neck, anti-monalizumab antibodies were found in 9 of the 25 patients (36%) (*51*).

Using a competitive panning strategy with NKG2A and NKG2C recombinant proteins, we successfully isolated and characterized fully human NKG2A inhibitors, 1B2 and 1B2-6 clones, through both phage and yeast display technologies. By contrast, the original murine predecessor of monalizumab (clone Z270) was generated from mice immunized with the CD94^bright^ NK cell clone SA260 (surface phenotype: CD3^−^CD16^+^, CD56^+^, NKp46^+^, NKp44^+^, p70/NKB1^+^, and CD94/NKG2A^+^) (*17*). Our investigation showed that monalizumab only bound to CD94/NKG2A heterodimer, not to the NKG2A monomer (fig. S1C). Targeting a cooperative conformational epitope formed by the CD94 and NKG2A complex allowed anti-NKG2A antibodies to efficiently inhibit HLA-E interaction as HLA-E also binds to a similar interface of the CD94/NKG2A heterodimer (*12*). In our mutagenesis studies, the serine residue at the 170 position (S170) near the connecting loop between alpha 2 helix and beta 3 sheet of the NKG2A domain was a critical residue for the selectivity of these three NKG2A antibodies versus NKG2C. However, substituting the serine to leucine (S170L) did not completely abolish binding for all NKG2A inhibitors, indicating that multiple residues cooperatively generate a distinct conformational epitope for the NKG2A-specific antibodies. At 167, 168, and 170 positions (based on human NKG2A numbering), NKG2 family receptors encode mismatched residues even in different species, while neighboring residues are identical or similar, except in mice. This region seems critical for recognizing self–HLA-E and shows different binding strengths to NKG2 family receptors (*12*, *52*).

NK cell activation is constrained by inhibitory receptors such as killer cell immunoglobulin-like receptors and NKG2A. The blockade of these inhibitory signals by antibodies releases the inhibitory signaling and leads to partial NK cell activation, as manifested by secretion of cytokines and GrzB (Figs. 2 and 5). Our WB analysis did not show a reduction in pSHP-1 levels following NKG2A blockade but instead a slightly reduced pCrk and increased pVav1 levels, suggesting that 1B2-6 may modulate NKG2A signaling through a SHP-1–independent c-Abl/pCrk pathway (*26*). However, further investigation is required to fully elucidate these signaling mechanisms. Regarding potential as an immunotherapeutic, NKG2A antibodies boosted NK cell–mediated ADCC in combination with either anti-EGFR, HER2, or CEACAM5 antibodies. On the other hand, NKG2A blocking antibodies alone failed to elicit NK cell–mediated cytotoxicity in the absence of tumor-directed antibodies in vitro during short-term culture (4 hours) or in vivo in a human cancer xenograft mouse model. This result is consistent with previous observations with monalizumab, which did not improve the survival rate of mice when used as a monotherapy (*13*). Although our antibodies disrupt NKG2A inhibitory signaling to partially activate NK cells, the cytolytic efficacy of NK cells against tumor cells appears to depend on other factors, including the formation and release of lytic granules, the epitope and binding properties of the tumor-targeting antibody, and involvement of other immune checkpoint receptors, or glycosylation status of membrane-bound proteins (*8*, *53*). For example, increased sialylated glycans on tumor cells limited NK cell–mediated cytotoxicity, including ADCC, by recruiting sialic acid–binding Ig-like lectin 7 (Siglec-7) (*54*, *55*). Further investigation is necessary to elucidate how NKG2A inhibitors affect CD8^+^ T cell cytotoxic function. In healthy donor PBMCs, the NKG2A^+^ population was below 2% in CD3^+^CD8^+^ T cells. By contrast, the expression level of NKG2A was significantly higher, up to 16%, in T cells isolated from cancer patient PBMC samples (fig. S11). Additional studies may address whether anti-NKG2A antibodies could elicit direct tumor cell killing by T cells and enhance complement-dependent cytotoxicity or antibody-dependent cellular phagocytosis by other immune cells.

Our results show that the NKG2A blocking antibodies can relieve tonic NKG2A-mediated repression but does not achieve to full NK activation and cytolysis. A key advance from the current work is that NKG2A has the potential to be used as a target for bispecific NK or T cell engagers. Through in vitro and in vivo assessments, we showed that BiNK constructs (aHER2 × aNKG2A and aEGFR × aNKG2A BiNK) recruit and activate both NK and CD8^+^ T cell cytotoxic functions. These results show that inhibitory receptors can also serve as targets for the immune cell engagers, consistent with that for the aHER2 × aPD1 T cell engagers that promotes contact between T cells and cancer cells (*56*). Our mechanistic studies indicated that induction of NKG2A internalization and partial blockade of NKG2A/CD94 signaling conferred by the anti-NKG2A arm of the BiNK may contribute to its activity. BiNK can assemble a cytolytic IS between NK and tumor cells, as demonstrated by multiple line of evidence including increased pVav1, cytokine induction, and cytolysis of tumor cells post BiNK treatment. Although BiNK activated NK cells, BiNK treatment appeared to increase pSHP-1 (fig. S5), suggesting that BiNK-mediated NK/tumor IS formation may promote NKG2A clustering and downstream pSHP-1 signaling while simultaneously recruiting other activating receptors within the synapse that drive NK cell activation. It is well-established that NK cell inhibitory and activating receptors coexist within the IS and collectively regulate NK cell function (*26*). Inhibitory receptors within the IS can modulate the distribution and microaggregation of activating receptors (*57*). Our results demonstrated that BiNK treatment increased the expression level of the activating receptor CD16A, a distinct property not observed with NKG2A antibodies alone. This up-regulation of CD16A likely contributes to the activating signals in the IS. Now, it cannot be excluded that BiNK-mediated IS recruits other activating receptors, such as NKp46, NKp30, and NKG2D, which may depend on stress-induced ligands on tumor cells.

We also found that the aHER2 × aNKG2A BiNK and pertuzumab IgG1 have distinct mechanisms of tumor cell killing. In our studies, the tumor-binding IgG1 antibody pertuzumab exhibited strong cytotoxicity in the in vitro assay using purified CD56^+^ NK cells as effector cells but did not regress tumor in the human PBMC-engrafted NSG mouse model bearing subcutaneous xenografts. By contrast, BiNK demonstrated antitumor efficacy both in vitro and in vivo. One likely explanation supported by in vivo NK and T cell depletion experiments is that BiNK engages both NKG2A^+^ NK cells and CD8^+^ cytotoxic T lymphocytes in vivo, whereas IgG1 antibodies primarily rely on CD16A^+^ NK cells for ADCC. As observed in our cell enumeration experiments, NK cells in the PBMC-humanized NSG model have a relatively short life span. NK-cell numbers declined over time, except in treatment groups showing effective antitumor activity, where NK activation likely promoted persistence, and CD8^+^ T cells expanded, reflecting T cell activation, potentially driven by both tumor-specific responses and low-level xenoreactivity. This sustained CD8^+^ T cell presence and activation may contribute to the observed in vivo efficacy of the aHER2 × aNKG2A BiNK.

Tumor-infiltrating NK (TINK) cells often exhibit an inert phenotype, characterized by high NKG2A expression and loss of CD16 with impaired cytotoxic capacity (*58*, *59*). PBMCs are predominantly cytolytic CD56dim/CD16^+^ cells with less numbers of CD56^+^CD16^−^ cells. The cytolytic CD16^+^ NK cells may express different chemokine receptors, which can influence their ability to migrate to and infiltrate tumors (*60*). The population that immigrates into the tumor microenvironment (TME) might favor CD16^−^/NKG2A^+^ cell types. This can lead to a functional shift away from the highly cytolytic, CD16^+^ phenotype toward less cytolytic phenotypes (*59*, *61*). Therefore, while IgG1 antibody pertuzumab primarily depends on CD16^+^ NK cells and may be limited by the scarcity or dysfunction of these cells in the TME, BiNK can effectively exploit NKG2A^+^ NK and CD8^+^ T cells, which are more prevalent and functionally accessible in the TME. This dual engagement likely underlies aHER2 × aNKG2A BiNK’s superior antitumor efficacy observed in vivo compared with pertuzumab. Given that NKG2A is broadly expressed on NK, NKT cells, and a subset of CD8^+^ T cells, NKG2A-directed engagers can engage and activate a broader range of effector immune cells than CD16A-based engagers.

In addition, targeting NKG2A by a BiNK molecule has potential to confer several advantages beyond existing NK or T cell engagers or mAbs: (i) It does not compete with patient-derived antibodies for binding to Fc gamma receptors to facilitate maintaining intrinsic humoral immunity, (ii) it does not interfere with the immune function of other activating receptors essential for antitumor immunity, and (iii) it could enhance the efficacy of other antitumor antibodies through NKG2A blocking ability.

In summary, we have successfully generated fully human NKG2A blocking antibody capable of activating NK cells and potentiating ADCC of tumor-binding antibodies. Furthermore, we have demonstrated a first-in-class BiNK molecule that co-opts an inhibitory receptor and a tumor-specific binder to enhance the cytotoxic potential of NK cells and T cells. These NKG2A-blocking reagents hold promise for further development and optimization, paving the way for clinical translation in solid tumors of high unmet need. By combining the blockade of an inhibitory receptor as the trigger for immune cell activation with tumor antigen-specific cell killing with the BiNK format, this work provides a framework for the development of more effective cancer immunotherapies.

## MATERIALS AND METHODS

### Preparation of soluble single-chain CD94 and NKG2A heterodimers

The single chain CD94 (residues 57 to 179) and either NKG2A (residues 113 to 233) and NKG2C (residues 111 to 231) were synthesized in Integrated DNA Technologies Inc. and then cloned into a pCATDS-Fc plasmid for the mammalian cell expression and purification via Not I and Asc I restriction enzyme sites. The constructed plasmid DNA was amplified from the *TOP10F Escherichia coli* strain in the presence of ampicillin (100 μg/ml) and prepared by a Midi DNA-prep kit (Macherey-Nagel, 740410) for the transient transfection. Purified DNA was complexed with PEI-Max (Polysciences, 24765-1) and supplied to the culture of the Freestyle human embryonic kidney cell line (Gibco, R79007). Seven days posttransfection, CD94/NKG2A-Fc and CD94/NKG2C-Fc were purified by affinity chromatography with protein A resin (Captiva, NC0997253). The elution of bound proteins to protein A was done by adding 50 mM glycine buffer pH 3.0, and then the buffer was changed to phosphate buffered saline (PBS) pH 7.4 by using PD-10 desalting column (GE, 45-000-148) for the storage. Protein purity was estimated in SDS-PAGE or SEC. The concentration of each protein was determined by NanoDrop spectrophotometer 2000C (Thermo Fisher Scientific, ND2000C).

### ICAT5 Fab library panning against CD94/NKG2A

Fully human Fab phage libraries were constructed previously (*28*). ICAT5 and ICAT5-1 phage libraries were preblocked with 3% bovine serum albumin (BSA) in PBS (w/v) for 1 hours at 25°C. Blocked phages were incubated with 10 nM biotinylated CD94/NKG2A-Fc for 1 hour at 25°C in the presence of 50 nM CD94/NKG2C-Fc. Bound phages were separated by streptavidin-coated magnetic beads (Invitrogen, 11-205-D) and washed 10 times with 1 ml of PBS (pH 7.4) containing 0.1% Tween 20 (w/v). The elution of bound phages was conducted by adding either 1 μM anti-NKG2A antibody or 5 nM HLA-E tetramer. For second and third rounds of panning, the reduced concentration of biotinylated CD94/NKG2A (5 and 1 nM, respectively) and increased competitor ratio of CD94/NKG2C were applied. After three rounds of panning, the binding of 192 individual clones was analyzed in ELISA, and then selected clones were sequenced after plasmid rescue.

### Purification of Fab and IgG (LALA-PG)

The plasmid of positive clones was transformed into *HB2151 E. coli* competent cells. Then, colonies were selected in ampicillin containing LB plate (final concentration of 100 μg/ml) for overnight in incubator at 37°C. The next day, a colony was inoculated in liquid LB + ampicillin media and cultured in 37°C shaking incubator. Isopropyl β-d-1-thiogalactopyranoside was added to the culture at optical density at 600 of between 0.4 and 0.6 corresponding to around 4 × 10^8^ cells/ml for a final concentration of 0.1 mM. The culture was relocated to a shaking incubator set at 30°C, 200 rpm, and incubated overnight. The next day, *E. coli* cells were harvested and resuspended in ^1^/_10_ volume of periplasm extraction buffer containing polymyxin B [0.5 mg/ml in PBS (pH 7.4)] and then incubated on the ice for an hour. The supernatant was collected by centrifugation at 12,000*g* for 10 min and then loaded into prepacked Ni–nitrilotriacetic acid resin. The bound Fab was eluted by adding 300 mM imidazole in PBS (pH 7.4), and then imidazole was removed by using a PD-10 desalting column. For IgG preparation, IgG-cloned plasmid DNA was transfected to human embryonic kidney–293F cells and expressed for 5 to 7 days posttransfection. Expressed IgG was purified by affinity chromatography with protein A resin. Bound IgG was eluted by adding 50 mM glycine buffer (pH 3.0), and then the storage buffer was changed to PBS (pH 7.4) by PD-10 desalting column.

### Variable region shuffled library construction for affinity maturation of 1B2 Fab

The 1B2 single-chain Fab (scFab) was subcloned into the N terminus of Aga2 in yeast surface-display plasmid pYCAT. Library genes encoding either VL (FR1-CDRL1-FR2-CDRL2-FR3-CDRL3-FR4) or partial VH (FR1-CDRH1-FR2-CDRH2-FR3) with homologous recombination sites were amplified by designed primers. cDNA isolated from healthy donor PBMCs was used as template DNA. Nhe I/BsI WI digested pYCAT plasmid was used for VL shuffled library, and Nhe I/Apa I digested plasmid was used for VH shuffling. Transformations were performed by using an EZ yeast transformation kit (Zymo Research, T2001). With the *EBY100* strain, sand yeast cells were selected directly in selective SD-CAA media [yeast nitrogen base (6.7 g/liter), casamino acids (5 g/liter), Na_2_HPO_4_ (5.4 g/liter), NaH_2_PO_4_·H_2_O (8.56 g/liter), and dextrose (20 g/liter) in deionized water] liquid media at 30°C overnight. The library screening was performed by three rounds of fluorescence-activated cell sorting (FACS) using FACS melody (BD Biosciences) against biotinylated CD94/NKG2A-Fc in the presence of a 10-fold molar excess of nonbiotinylated CD94/NKG2C-Fc as a competitor. For inducing expression of scFab on yeast surface, 1 × 10^9^ yeast cells were cultured in SG-CAA media (used galactose instead of dextrose for SD-CAA) at 30°C for 16 hours, then induced cells were washed with diluted autoMACS buffer (Miltenyi, 130-091-221) in Dulbecco’s PBS (DPBS) (pH 7.4). The cell surface expression and antigen binding level were determined by anti-kappa fluorescein isothiocyanate (Invitrogen, A18854) and streptavidin phycoerythrin (PE) (Invitrogen, S866) or Alexa Fluor 647 conjugated (Invitrogen, S21374).

### Binding of Fab or IgG in ELISA

The binding and specificity of Fab or IgG to CD94/NKG2A or 2C-Fc were analyzed through indirect ELISA. Briefly, Fc-fused proteins were coated on a 96-well plate (Corning, 3690) at 200 ng per well (50-μl volume) in PBS for 2 hours at room temperature. Blocking was carried out with 3% BSA in PBS overnight at 4°C. The next day, various Fab or IgG concentrations were added to antigen-coated plates and incubated for 1 hour at 25°C. After three washes, anti-FLAG mouse antibody (M2 clone)–horseradish peroxidase (HRP) conjugated (1:3000 dilution; Sigma-Aldrich, A8592) or anti-human kappa goat antibody-HRP conjugated (1:3000 dilution; Invitrogen, A18853) was added to detect binding of either Fab or IgG. The same volume of 3,3′,5,5′-tetramethylbenzidine (TMB) (Thermo Fisher Scientific, PI34028) was added as a substrate, and then the enzymatic reaction was stopped by adding 2 N sulfonic acid. Biotinylated HLA-E *01:01 (peptide sequence: VMAPRTLVL) was purchased from MBL International, and it was complexed with streptavidin-HRP in a 4:1 molar ratio. Then, 100 pM tetramer was used for competitive ELISA.

### Biolayer interferometry

The affinities of antibodies were analyzed using the BLI (BLItz, ForteBio, USA). Biotinylated scCD94/NKG2A-Fc or biotinylated HER2 was mounted on an SA Biosensor (Sartorius, 18-5019) for 2 min and equilibrated with PBS (pH 7.4) to establish baselines. Then, the anti-NKG2A antibodies (25, 50, 100, or 200 nM) were used for association for 2 min, and then the antibody was allowed to dissociate in PBS for 6 min. For the simultaneous binding analysis of BiNK antibodies to NKG2A and EGFR/HER2, biotinylated scCD94/NKG2A-Fc was mounted on the SA Biosensor for 1 min and equilibrated with PBS (pH 7.4) to establish baselines for 2 min. Then, the BiNK antibodies (100 nM) were used for association for 2 min and then equilibrated with PBS (pH 7.4) for 2 min. Then, the HER2-his or EGFR-his was used for association for 2 min and then was allowed to dissociate in PBS for 2 min. The background was established by running PBS instead of antibody in the association process, and the background curve was subtracted from the antibody sensorgram. The *k*_on_ and *k*_off_ were derived from sensorgram fittings and used for *K*_D_ calculation.

### Size exclusion chromatography

The ÄKTA pure chromatography system (GE Healthcare) was used. The column (Superdex 200 Increase 10/300 GL) was calibrated with protein molecular mass standards of thyroglobulin [relative molecular mass (*M*_r_) of 669,000 kDa], ferritin (*M*_r_ of 440,000 kDa), aldolase (*M*_r_ of 158,000 kDa), conalbumin (*M*_r_ of 75,000 kDa), ovalbumin (*M*_r_ of 44,000 kDa), carbonic anhydrase (*M*_r_ of 29,000 kDa), and ribonuclease A (*M*_r_ of 13,700 kDa). About 200 μl of filtered proteins (1 mg/ml) in PBS was used for analysis. Protein was eluted by DPBS buffer at a flow rate of 0.45 ml/min.

### Cell lines

NCI-H660, Du145, A549, H2030, Farage, and 293T cells were purchased from American Type Culture Collection (ATCC). Du145 cells were maintained in Eagle’s minimum essential medium (EMEM, ATCC) supplemented with 10% (v/v) FBS (Gibco) and 1% penicillin-streptomycin (P/S, Gibco). A549, H2030, and Farage cells were cultured RPMI 1640 (ATCC) supplemented with 10% (v/v) FBS and 1% P/S. 293T cells were maintained Dulbecco’s modified eagle’s medium (DMEM, Gibco) supplemented with 10% (v/v) FBS and 1% P/S. NCI-H660 cells were cultured in RPMI 1640 supplemented with 5% FBS, 1X insulin-transferrin-selenium (ITS-G, Gibco), 10 nM hydrocortisone, extra 2 mM l-glutamine, and 1% P/S. Du145-CEACAM5 cells were previously constructed and described (*28*). Primary NK cells were cultured with magnetic-activated cell sorting (MACS) basal NK media with supplementary (Miltenyi Biotec, 130-114-429), 10% (v/v) human serum (Sigma-Aldrich), and human IL-2 (hIL-2) (50 IU/ml; Miltenyi Biotec).

### Flow cytometry analysis

Primary NK cells were enriched from normal human PBMCs (Zenbio Inc.) using an NK cell isolation kit (Miltenyi Biotec, 130-092-657). The purity of isolated NK cells was confirmed by staining of allophycocyanin (APC)–conjugated anti-human CD56 antibody and PE-conjugated anti-human CD16 antibody. The HLA-E expression of target cells was determined by staining of PE-conjugated anti–HLA-E antibody. To determine the NKG2A^+^ population of primary NK cells, cells were stained with Alexa488 (AR488)–conjugated anti-NKG2A IgG1. To confirm the cell surface binding of antibodies (mona-IgG, 1B2 IgG, and 1B2-6 IgG), cells were treated with antibodies (100 or 10 nM) for 1 hour at 4°C and then stained Alexa647-conjugated goat anti-human IgG (Invitrogen, A21445) for 0.5 hours at 4°C. For investigation of antibody internalization in NK cells, pH-sensitive dyes were conjugated to antibodies using the pHAb reactive dye (Promega, G9841). The enriched NK cells were incubated with hIL-2 (50 IU/ml) and treated with the indicated pHAb-conjugated antibodies (100 nM) for 24 hours at 37°C, either alone or in cocultured with A549 cells. For the gating of NK cells in the coculture system with H2030 cells, APC-conjugated anti-human CD45 antibody was used. Monoclonal antibodies specific for CD56 (12-0567-42), CD16 (12-0168-42), CD159a (130-113-566), CD45 (17-0459-42), and HLA-E (12-9953-42) were purchased from Thermo Fisher Scientific. For intracellular staining, GolgiPlug containing brefeldin A (Invitrogen, 00-4506-51) was added for the final 4 hours of culture to inhibit protein transport from the endoplasmic reticulum to the Golgi apparatus. All intracellular staining was performed using a BD cytofix/cytoperm kit (BD Biosciences, BD554714). Monoclonal antibodies specific for IFN-γ (12-7319-42) and GrzB (12-8896-42) were purchased from Thermo Fisher Scientific. Durvalumab (anti–PD-L1 antibody, A2013), cetuximab (anti-EGFR antibody, A2000), and pertuzumab (anti-HER2 antibody, A2008) were purchased from Selleck Chemicals. Data were acquired using the flow cytometry BD LSR II (San Jose, CA) and analyzed with FlowJo 10.7.1 (Tree Star).

### Functional assay with peripheral human NK cells

The purified human NK cells from PBMCs were used as effector cells. NK cells were incubated with target cell line (H2030) (E:T ratio, 5:1) in the presence of hIL-2 (50 IU/ml) for 24 hours at 37°C. Cells were dispensed into 96-well plates with or without antibodies (mona-IgG, 1B2 IgG, 1B2-6 IgG, and durvalumab) at 100 nM for 24 hours at 37°C. After treatment of antibodies, cells were fixed and intracellular stained with PE-conjugated anti-human IFN-γ antibody and PE-conjugated anti-human GrzB antibody. Cells were washed twice and analyzed using the BD LSR II flow cytometry.

### In vitro cytotoxicity assay

The ADCC or cell killing activity of effector cells incubated with anti-NKG2A antibodies with LALA-PG mutation was measured through the release of cytosolic lactate dehydrogenase (LDH) from dead target cells using LDH-Glo cytotoxicity assay kit (Promega, J2381). The purified NK cells from normal PBMCs (Zenbio Inc.) were used as effector cells and were incubated with A549, H2030, 293 T, NCI-H660, Du145, or Du145-CEACAM5 cells (1 × 10^4^ cells per well in 96-well plate) as target cells at E:T ratio, 5:1 in the presence of the indicated antibodies for 4 hours at 37°C. CD8^+^ T cells were isolated from normal human PBMCs (Thermo Fisher Scientific, 11333D) and used only in A549 coculture assays. The percent cytotoxicity was calculated as previously described (*28*).

### Western blot

Total proteins were extracted from NK cells by radioimmunoprecipitation assay buffer containing 1× protease inhibitors (Thermo Fisher Scientific, 89901 and 78441). A 20 μg of total proteins of each sample was loaded into a 4 to 12% bis-tris mini protein gel (Thermo Fisher Scientific, NP0321BOX). Proteins were then transferred to a 0.2-μm polyvinylidene difluoride membrane (Bio-Rad, 12023954). The membrane was blocked in 5% nonfat milk in tris-buffered saline (TBS) for 1 hour at room temperature, followed by incubation with Vav-1 (Cell Signaling Technology, 2502), pVav-1 (Tyr^174^, Thermo Fisher Scientific, PA5-78220), Crk (Thermo Fisher Scientific, MA5-15891), pCrk (Tyr^221^, Thermo Fisher Scientific, MA5-35895), pSHP-1 (Tyr^564^, Cell Signaling Technology, 8849), and β-actin (Thermo Fisher Scientific, MA1-140) antibody at 1:1000 dilution at 4°C overnight. After three washes with tris-buffered saline with Tween 20 (TBST) buffer, the membranes were incubated with anti-rabbit or mouse HRP secondary antibody at 1:4000 dilution for 1 hour at room temperature. The membranes were visualized by using ECL Western blot substrate (Thermo Fisher Scienctific, 32106) and imaged by a ChemiDoc XRS+ Imaging System (Bio-Rad).

### In vivo study

All studies were approved by the University of Pittsburgh Institutional Animal Care and Use Committee (protocol no. 22101538). For PK experiments, female BALB/c mice (6 weeks old) received a single injection (at 10 mpk) of various antibodies in a total volume of 200 μl via the tail vein. At the time points, 1, 4, 8, 18, and 24 hours and on days 2, 4, 7, 14, and 21, blood samples were taken from the tail vein or retro-orbital sinus of CO_2_-anesthetized mice (*n* = 3 per time point). Concentrations of the antibody in the serum samples were determined by an ELISA (*62*). The first value (at 30 min) was set to 100% for comparison. PK parameters [distribution phase serum half-life (*T*_1/2_α) and elimination phase serum half-life (*T*_1/2_β)] were calculated by two-phasic nonlinear regression analysis in the GraphPad Prism software. For the tumor growth inhibition (TGI) experiment, NOD-scid IL2Rgnull mice (NSG mice, 6 weeks old, female, the Jackson Laboratory) were engrafted subcutaneously (right flank) with A549 (5 × 10^6^ cells per mice) in 200 μl of a 1:1 mixture of PBS/Matrigel. When the tumors were 150 mm^3^, human PBMCs, NK cell–depleted PBMCs, or CD8^+^ T cell–depleted PBMCs (1 × 10^7^ cells per mice) were intravenously injected via tail vein once a week for two consecutive weeks. Mice were intravenously treated with pertuzumab (5 mg/kg), 1B2-6 (10 mg/kg), and BiNK (anti-HER2 × anti-NKG2A, 10 mg/kg), twice a week, four times, via tail vein (*n* = 6 per group). Tumor volume was measured by two-dimensional measurements with a caliper and calculated according to the formula *V* = 0.5 × length × (width)^2^. TGI by 1B2-6, BiNK, pertuzumab, 1B2-6 + pertuzumab, and BiNK + pertuzumab compared to that by vehicle was determined on the last day of the study according to the formula: TGI (%) = [1 − (*V*_f_^treated^ − *V*_i_^treated^)/(*V*_f_^control^ − *V*_i_^control^)] × 100, where *V*_f_ is the final mean tumor volume in the treated group and *V*_i_ is the initial mean tumor volume in the control group (vehicle). For NK/T cell population analysis, blood was drawn at 2, 4, and 6 weeks for most groups, except for the vehicle group at 5 weeks, the 1B2-6 group at 5.5 weeks, and the pertuzumab group at 5.5 weeks. PBMCs were isolated using Ficoll density gradient centrifugation (GE Healthcare, GE17-1440-02). Animals were euthanized when the tumor volume reached >1.5 cm^3^.
