# Supplementary material for: Discovery and preclinical evaluation of monoclonal antibodies and bispecific engagers targeting the NKG2A inhibitory receptor
Source: Sci Adv. 2026 Feb 4;12(6):eadu0690. doi: 10.1126/sciadv.adu0690 (PMC12871476; doi:10.1126/sciadv.adu0690)
Supplement: Supplementary file 1 — Figs. S1 to S11 [file sciadv.adu0690_sm.pdf]

Science Advances
AAAS

Supplementary Materials for

Discovery and preclinical evaluation of monoclonal antibodies and bispecific engagers targeting the NKG2A inhibitory receptor

Seungmin Shin *et al.*

Corresponding author: John W. Mellors, jwm1@pitt.edu; Dimiter S. Dimitrov, dimitrov666666@yahoo.com; Wei Li, liwei171@pitt.edu; Du-San Baek, Du-San.Baek@glpg.com

Sci. Adv. **12**, eadu0690 (2026)
DOI: 10.1126/sciadv.adu0690

This PDF file includes:

Figs. S1 to S11

Figure. S1

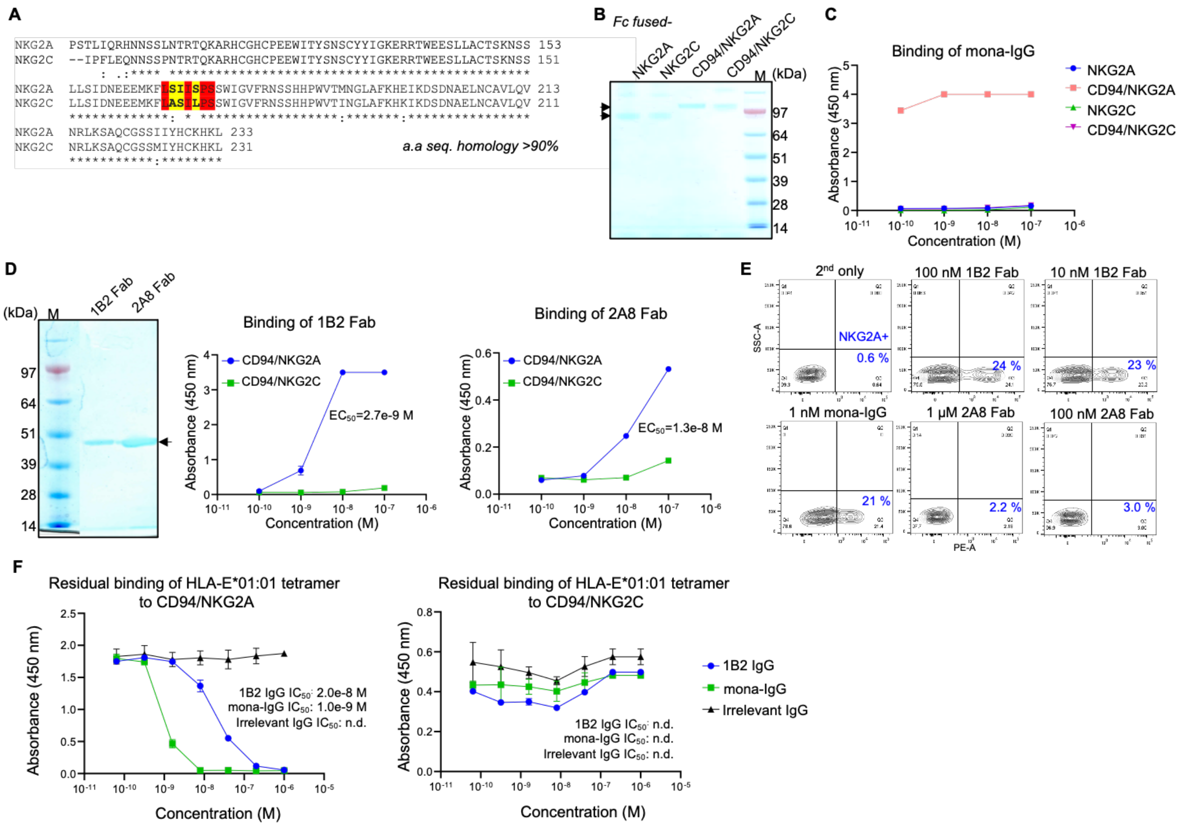

Figure S1. Discovery of NKG2A binding antibody, 1B2 clone. (A) Comparison of primary sequences of NKG2A and 2C. The mismatched residues between NKG2A and 2C at 167, 168 and 170 positions of NKG2A are colored as yellow. (B) SDS-PAGE gel for purified Fc-fused NKG2A or 2C with or without CD94. Arrows indicate corresponding band for four recombinant proteins (MW: Fc-fused NKG2A or 2C is 84 kDa, and Fc-fused CD94/NKG2A or 2C is 109 kDa.) (C) Indirect ELISA result indicating CD94 is essential domain for the binding and specificity of monalizumab analog (mona-IgG). (D) Specificity and binding of purified 1B2 and 2A8 Fab against CD94/NKG2A or 2C in ELISA. (E) Representative density plots depicting percentages of primary NK cells expressing CD94/NKG2A labeled by mona-IgG, 1B2 Fab, and 2A8 Fab in flow-cytometry after treatment of each antibody at indicated concentration for an hour. (F) Competitive ELISA experiment with sHLA-E tetramer demonstrating that 1B2 IgG and mona-IgG selectively block HLA-E binding to CD94/NKG2A. ELISA results are representative of two replicates and data are presented as mean± s.d.

Figure. S2

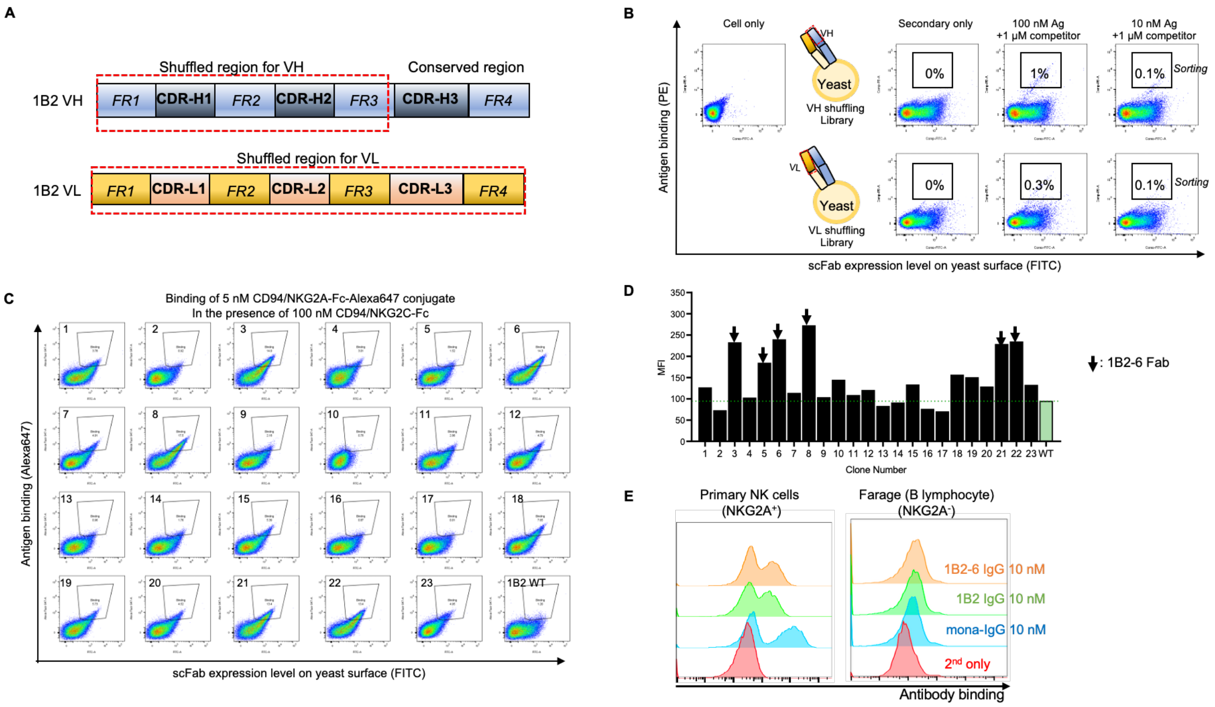

Figure S2. Affinity maturation of 1B2 Fab by using yeast surface display. (A) Schematic depicting diversification regions in VH and VL of 1B2. (B) Flow cytometry analysis of initial VH and VL shuffling libraries to confirm binders are in the constructed scFab library pool and to determine sorting gates. (C) Representative density plots showing scFab expression and antigen binding for individual clones after several rounds of sorting. (D) Bar-graph indicating mean-fluorescence-intensity (MFI) in Panel C. (E) Binding of mona-IgG, 1B2 IgG, and 1B2-6 IgG antibodies at 10 nM concentration to NKG2A on primary NK cells (left) and the NKG2A negative Farage B cell line (right).

Figure. S3

A

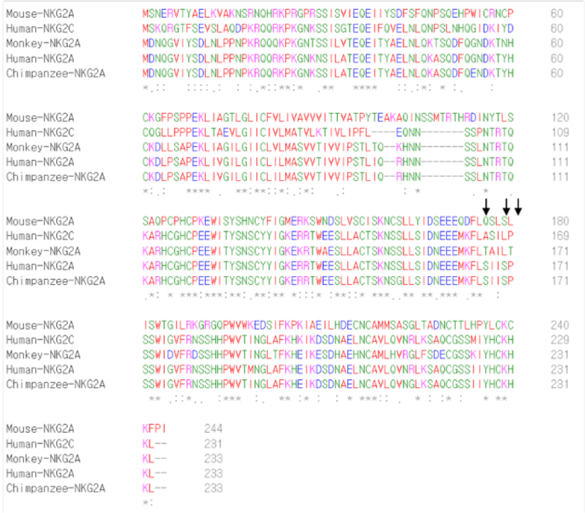

B

Type of receptor (species)	Name of receptor	Residue at indicated position (arranged to human NKG2A)		
		167	168	170
Inhibitory receptor (Homo sapiens)	NKG2A	Ser (S)	Ile (I)	Ser (S)
	NKG2B	Ser (S)	Ile (I)	Ser (S)
Activating receptor (Homo sapiens)	NKG2C	Ala (A)	Ser (S)	Leu (L)
	NKG2E	Ala (A)	Ser (S)	Leu (L)
	NKG2H	Ala (A)	Ser (S)	Leu (L)
Inhibitory receptor (Chimpanzee)	NKG2A	Ser (S)	Ile (I)	Ser (S)
Inhibitory receptor (Rhesus macaque)	NKG2A	Thr (T)	Ala (A)	Leu (L)
Inhibitory receptor ()	NKG2A	Gln (Q)	Ser (S)	Ser (S)

C

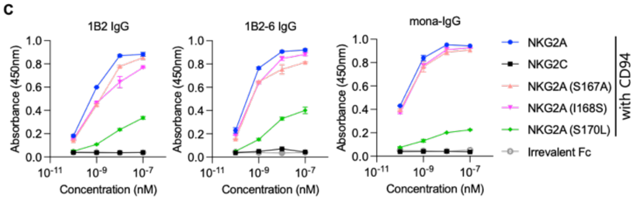

Figure S3. Primary sequence comparison of the NKG2A inhibitory receptor across different species. (A) Sequence alignment of NKG2A inhibitory receptors in human, mouse, monkey, and chimpanzee. Human NKG2C sequence is included for comparison. Arrows indicate potentially critical epitopes for NKG2A inhibitory antibody binding. (B) Residues at 167, 168, and 170 positions of human NKG2A and corresponding residues in NKG2A family member receptors in different species. Green color indicates hydrophilic residues and red color highlights hydrophobic residues. (C) ELISA experiment for the epitope mapping of three NKG2A inhibitors with mutant proteins of CD94/NKG2A. Each residue at 167, 168, and 170 positions of NKG2A was replaced by the indicated NKG2C residue.

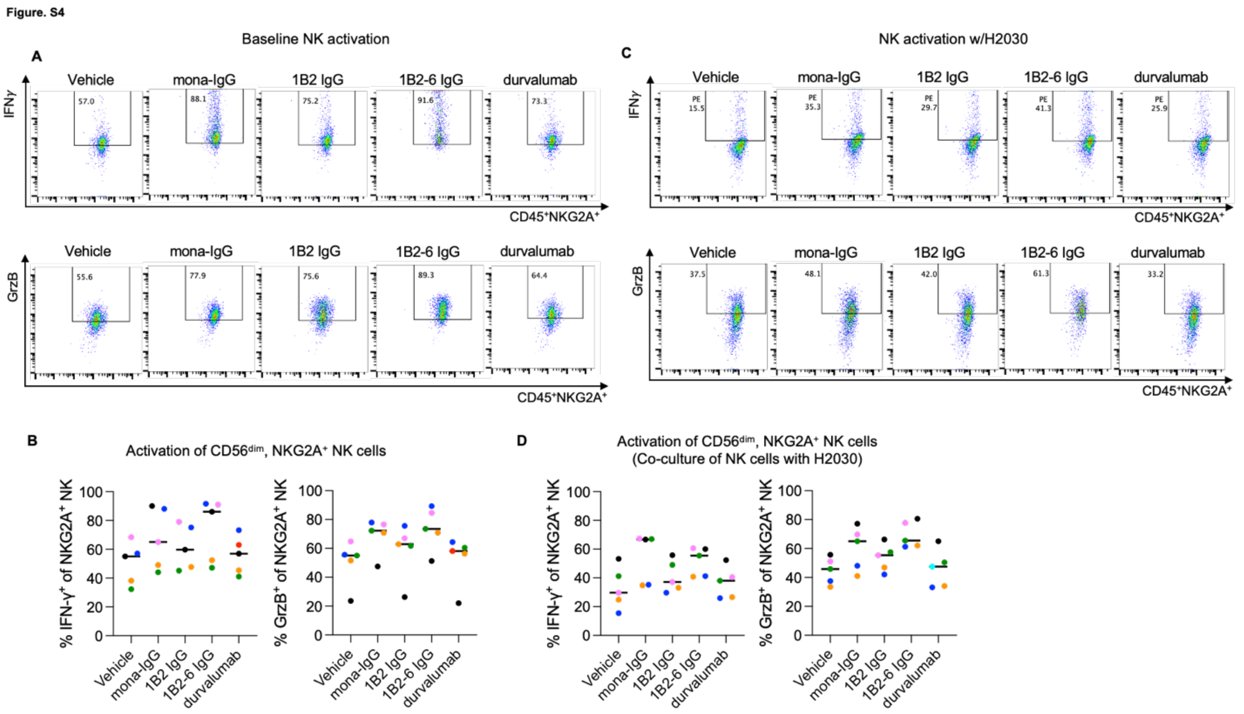

Figure S4. Assessment of IFN-γ and GrzB levels in primary NK cells by flow-cytometry. (A and C) Representative density plots indicating the percentage of cells expressing IFN-γ and GrzB to assess activation of primary NK cells (CD56^{dim}, NKG2A^{+}) cultured alone (A) or co-cultured with H2030 cancer cells (C) after treatment with anti-NKG2A antibody compared with the anti-PD-L1 antibody, durvalumab, for 24 hours. (B and D) Corresponding raw data before normalization, representing values shown in Figure 2A and 2B, respectively.

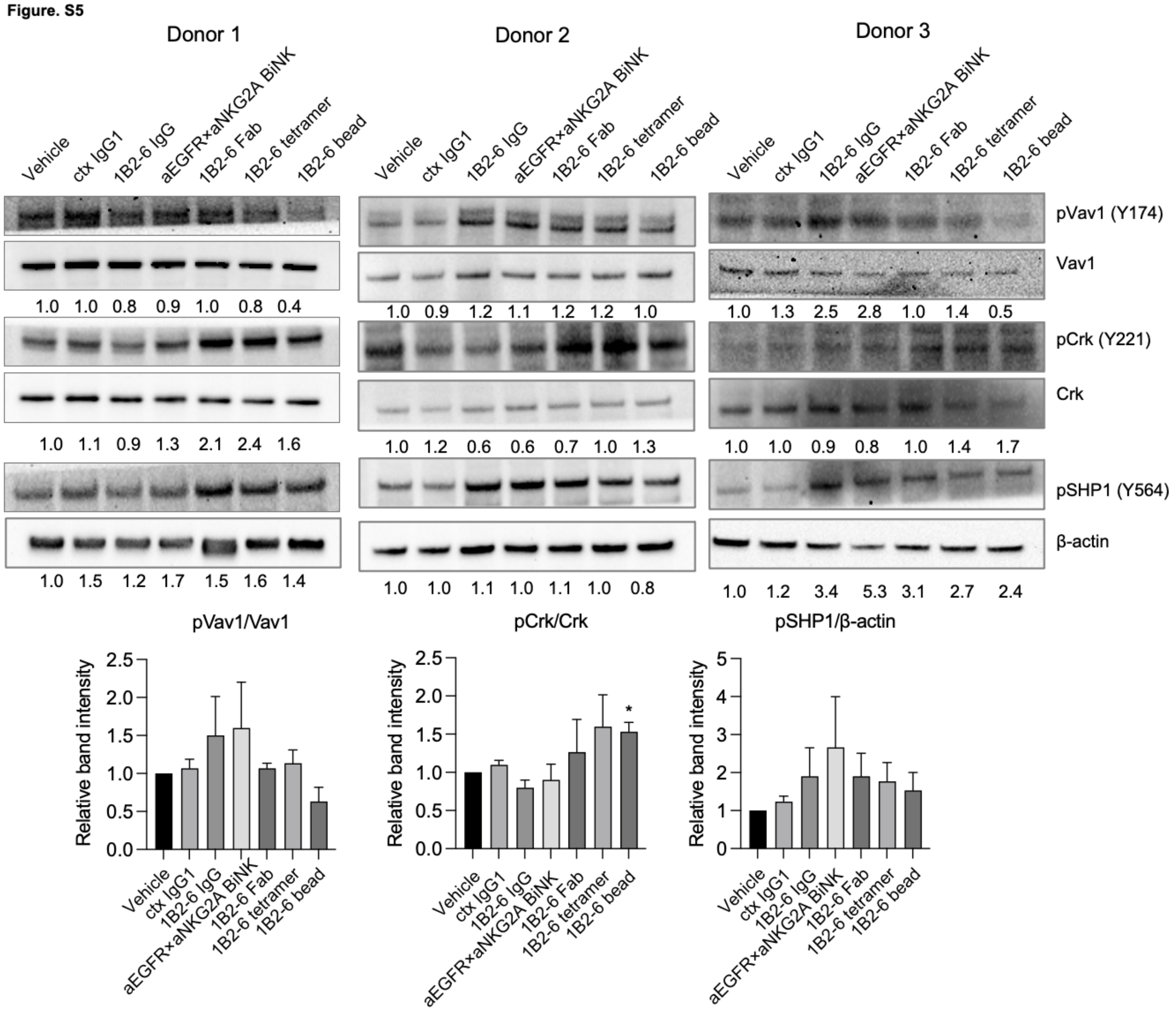

Figure S5. NKG2A downstream signaling in primary NK cells. Phosphorylation level of Vav1 (Y174), Crk (Y221), and SHP-1 (Y564) in NK cells from three independent healthy donors after treatment with anti-NKG2A antibodies. Below, values of pVav1, pCrk, and pSHP1 are shown to be normalized to total Vav-1, Crk, and β-actin, respectively, and expressed relative to the vehicle control.

Figure. S6

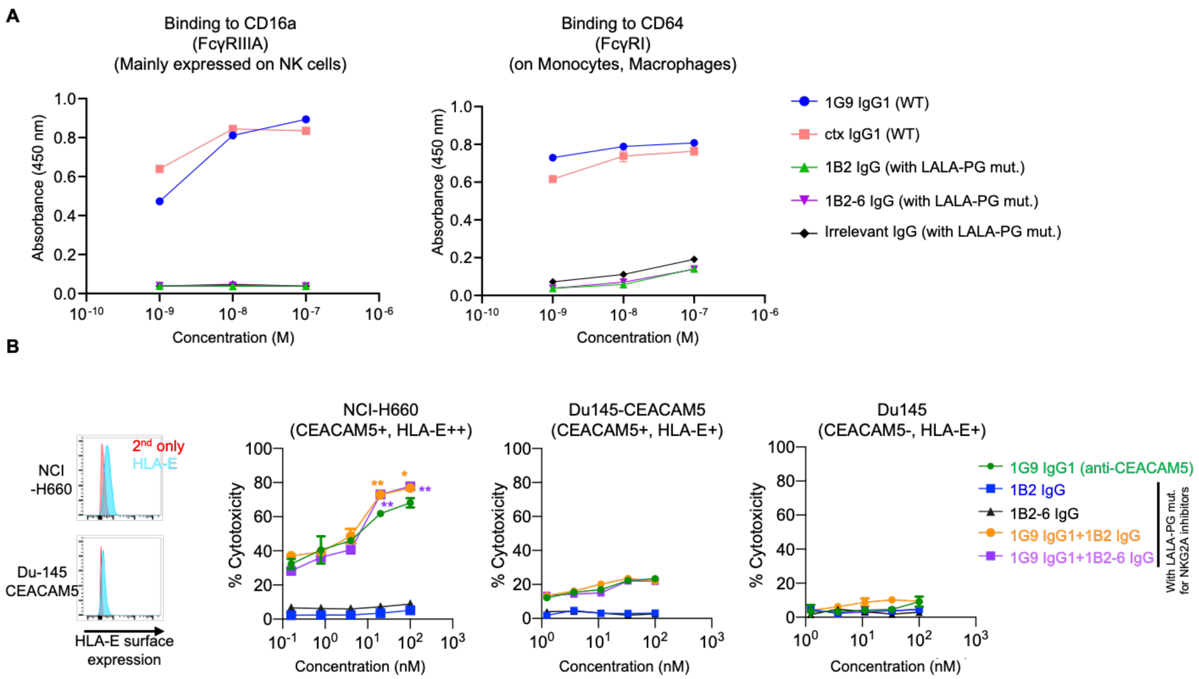

Figure S6. Binding of anti-NKG2A antibodies to Fc receptors (CD16a and CD64) and their enhancement of CEACAM5 antibody-mediated ADCC in vitro. (A) With L234A, L235A, and P329G mutations in human IgG1 Fc region (LALA-PG), anti-NKG2A antibodies show decreased binding to both CD16a and CD64 in ELISA, compared to IgG1 wild-type (WT). (B) Cell surface HLA-E expression level of NCI-H660 and Du145-CEACAM5 cells (left panel). ADCC activity of anti-CEACAM5 hIgG1 1G9 with 1B2 IgG or 1B2-6 IgG in prostate cancer NCI- H660, Du145-CEACAM5, and Du145 cells (right graphs). LDH release assay results in presence of primary NK cells from healthy donor PBMCs. E:T ratio, 5:1. Significance was determined by unpaired two-tailed student's t-test. **P* < 0.05, ***P* < 0.01, ****P* < 0.001, *****P* < 0.0001 vs. 1G9 IgG1 single treatment.

Figure. S7

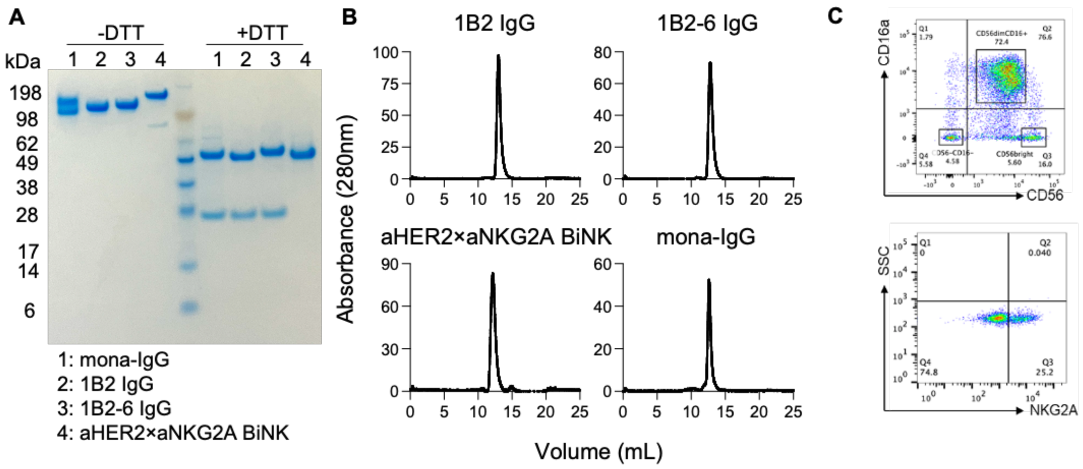

Figure S7. Assessment of biophysical properties of NKG2A antibodies and characterization of NK cell subsets from human PBMCs (A) SDS-PAGE analysis of purified NKG2A antibodies. (B) Size-exclusion chromatography (SEC) profiles of NKG2A antibodies. (C) The differential proportions of NKG2A^{+} and CD56^{dim}CD16A^{+} subsets in the isolated NK cell population derived from human PBMCs.

Figure. S8

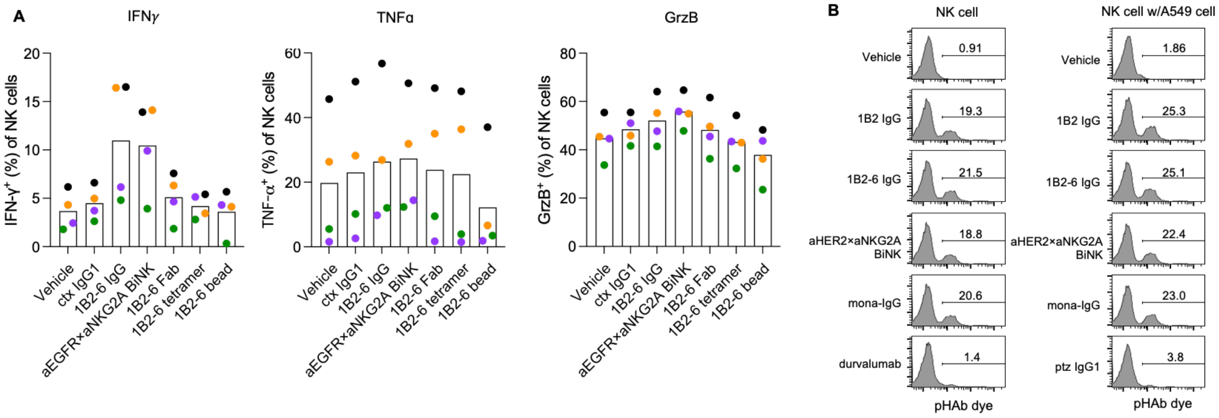

Figure S8. NK cell activation by NKG2A antibodies of different binding valency and NKG2A downregulation. (A) Percentage of IFN-γ, TNF-α, and Granzyme B (GrzB) positive NK cells after antibodies treatment with different NKG2A binding valency (monovalent, bivalent, tetravalent, or multivalent) in co-culture with A549 cells. Raw data before normalization, corresponding to Figure 5A, are shown. (B) Representative histograms of NKG2A downregulation and pH sensitive dye (pHAb)-conjugated antibody internalization in NK cells alone (left) or co-cultured with A549 cells (right). Corresponds to the representative FACS plots in Figure 5B. ELISA results are representative of two replicates and data are presented as mean± s.d.

Figure. S9

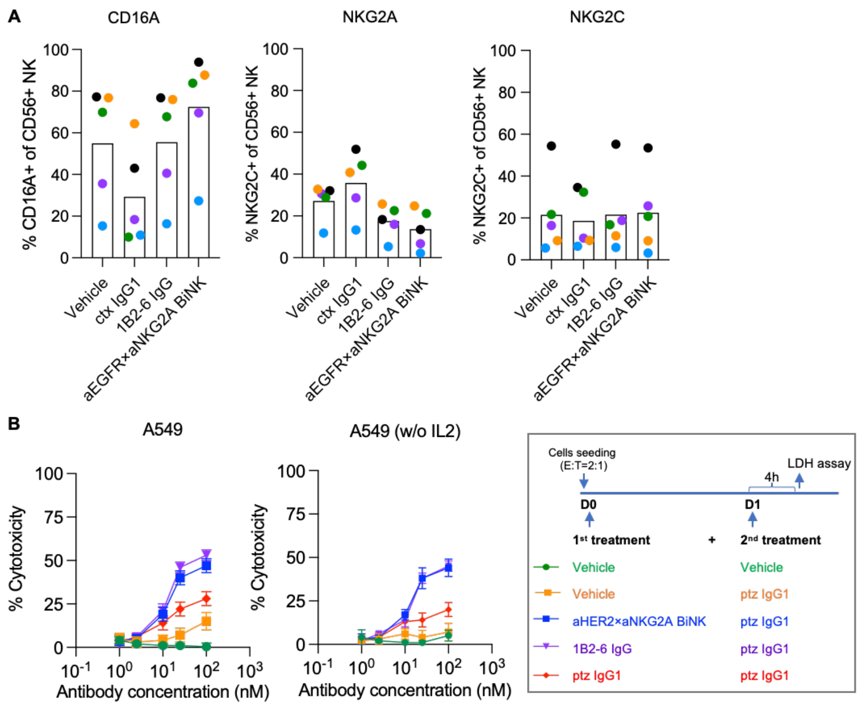

Figure S9. NK cell receptor modulation by BiNK antibody. (A) The CD16A$^+$, NKG2A$^+$, or NKG2C$^+$ populations (%) of CD56$^+$ NK cells after treatment with antibodies (100 nM) and 50 IU/ml IL-2 in co-culture with A549 cells (E:T ratio 5:1) for 24 hours. Raw data before normalization, corresponding to Figure 6A, are shown. (B) The bar graphs showing the cell-killing activity of aHER2 × aNKG2A BiNK in combination with anti-HER2 IgG1 pertuzumab (ptz IgG1) in A549 cells in the presence of primary NK cells with 50 IU/mL IL-2 (left panel) and without IL-2 (middle panel). E:T ratio, 2:1. Right, schematic figure of antibodies treatment schedule. (A) Each symbol represents the value obtained from individual healthy donors. Significance was determined by one-way ANOVA with the Tukey's post hoc test, *$P < 0.05$, **$P < 0.01$, ***$P < 0.001$. ****$P < 0.0001$.

Figure. S10

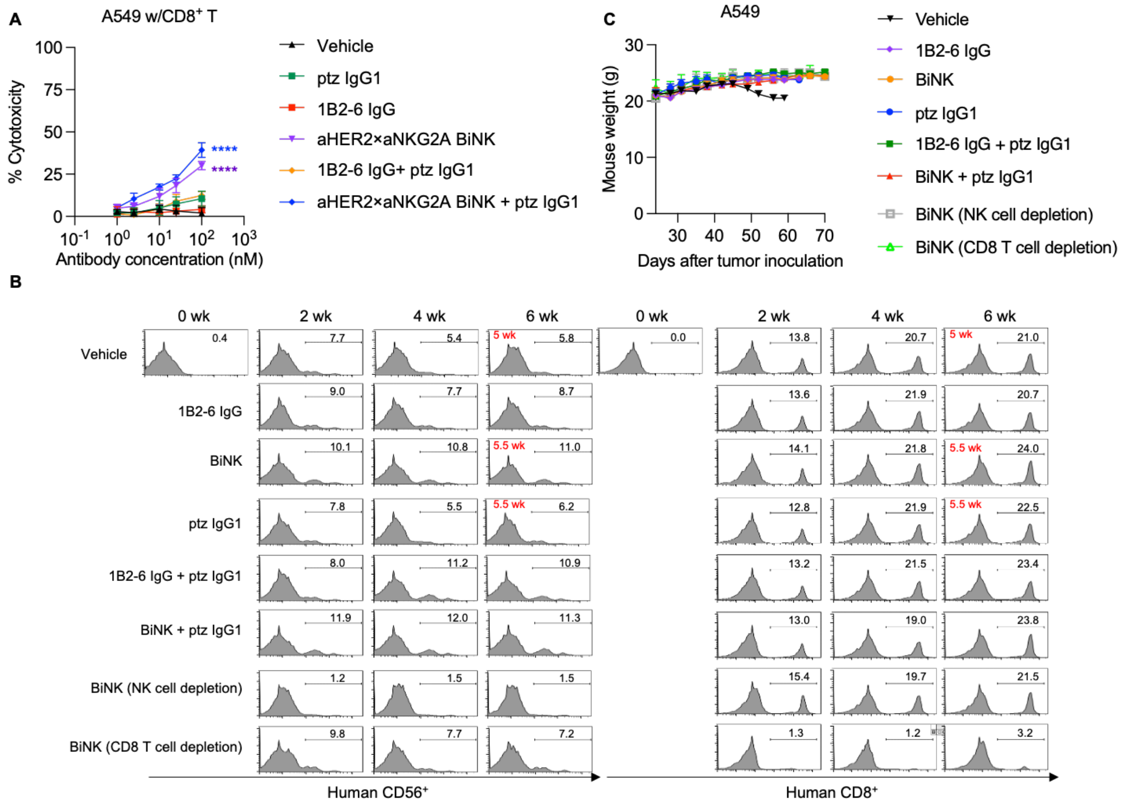

Figure S10. NK and CD8⁺ T cell dynamics and body weight during NKG2A inhibitor treatment (A) Cell killing activity of antibodies against A549 cells in the presence of CD8⁺ T cells (E:T ratio, 5:1), for 4 hours. (B) Representative histograms of human NK (left panel) and CD8⁺ T cell (right panel) populations in mouse peripheral blood at the indicated time points after human PBMC injection. Blood was drawn at 2, 4, and 6 weeks for most groups except for vehicle at 5 weeks, 1B2-6 at 5.5 weeks, and pertuzumab (ptz IgG1) at 5.5 weeks. (C) Mouse body weight measured during the treatments of NKG2A antibodies post human PBMC engraftment. Error bars represent the mean ± SD (n = 6 per group). (A) Results are shown as the mean ± SD for representative data from three independent experiments.

Figure. S11

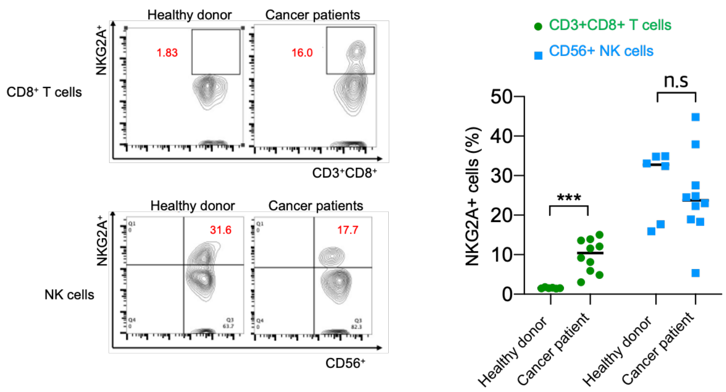

Figure S11. NKG2A expression in NK and CD8⁺ T cell population from PBMC of healthy donor and cancer patients. Representative density plot (left panel) and median percentage (right panel) of NKG2A positive human CD8⁺ T cell and NK cell in PBMC of healthy donor (n = 6) and cancer patients (n = 10). ***$P < 0.001$, vs. healthy donor. n.s means not significant.
